# H_2_ S‐Mediated GH3.1 Persulfidation Regulates IAA Homeostasis to Enhance Nodulation Formation and Nitrogen Fixation in *Robinia pseudoacacia*

**DOI:** 10.1111/mpp.70145

**Published:** 2025-10-03

**Authors:** Weiqin Zhang, Huaping Cheng, Xiaowu Yan, Bingyu Suo, Shiming Wen, Wuyu Liu, Gehong Wei, Juan Chen

**Affiliations:** ^1^ State Key Laboratory of Crop Stress Biology in Arid Areas, Shaanxi Key Laboratory of Agricultural and Environmental Microbiology College of Life Science, Northwest A&F University Yangling Shaanxi People's Republic of China

**Keywords:** Gretchen Hagen 3.1 (GH3.1), hydrogen sulphide (H_2_S), indeterminate nodule, indole‐3‐acetic acid (IAA), nitrogen fixation, *Robinia pseudoacacia*

## Abstract

Hydrogen sulphide (H_2_S), a gaseous signalling molecule, plays a multifaceted role in plant physiology by enhancing adaptability to environmental stresses. However, the regulatory mechanism of symbiotic nitrogen (N) fixation by H_2_S in indeterminate nodules of woody legumes remains unclear. In this study, we investigated the mechanism by which H_2_S promotes nodulation and N fixation in the woody legume *Robinia pseudoacacia* . Exogenous H_2_S significantly enhanced rhizobium infection, nodule formation and nitrogenase activity, demonstrating its positive role in the symbiotic process. Transcriptomic analysis of roots and nodules revealed that H_2_S signalling modulates auxin metabolism, particularly through the regulation of indole‐3‐acetic acid (IAA) homeostasis. H_2_S was found to promote free IAA accumulation and reduce IAA conjugation (IAA‐Asp and IAA‐Glu). Further investigation revealed that H_2_S directly targets GH3.1, a key IAA‐amido synthetase responsible for IAA conjugation. Specifically, H_2_S mediated persulfidation at Cys304 of GH3.1, inhibiting its enzymatic activity and preventing IAA inactivation. This modification was confirmed by LC–MS/MS, UPLC‐ESI‐MS/MS and site‐directed mutagenesis. This post‐translational modification maintained active IAA levels, facilitating early nodule development. These findings highlight the active role of H_2_S in regulating IAA homeostasis, thereby enhancing indeterminate nodule formation and N fixation through persulfidation of the Cys304 residue of GH3.1 in *R. pseudoacacia* .

## Graphical abstract

H_2_S persulfidates GH3.1 at Cys304, reducing its activity, sustaining IAA levels, triggering H_2_S‐IAA feedback, enhancing nodulation gene expression, boosting nodule formation, nitrogen fixation and *Robinia pseudoacacia* growth.

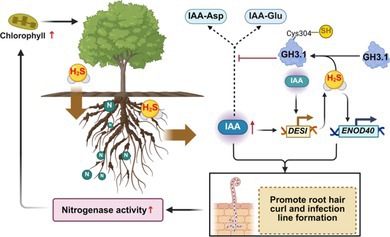

## 1 Introduction

Soil fertility and ecological sustainability are closely linked to the nitrogen (N) cycle (Gruber and Galloway 2008). N is essential for all life and constitutes about 70% of the atmosphere, yet its conversion into usable forms for plants and animals is challenging due to its stable triple bond. This limited accessibility hinders sustainable ecosystem development (Zehr and Capone 2020). However, studies show that crops effectively absorb only a tiny fraction of applied N fertiliser, with most lost through soil erosion, leading to resource wastage and negative impacts on soil quality and biodiversity (Haskett et al. 2021). Biological N fixation is a vital process in which microorganisms, including free‐living, associative and symbiotic forms, convert inorganic N compounds into ammonia, thereby addressing various low‐availability N sources (Olivares et al. 2013; Pankievicz et al. 2019; Soumare et al. 2020). Microorganisms utilise carbon sources provided by the host for synergistic interactions and fix N ranging from 50 to 70 teragrams (Tg) per year (Pankievicz et al. 2019; Soumare et al. 2020). Therefore, the improvement of the symbiotic N fixation capability in organisms is critical for environments with low N utilisation efficiency.

The symbiotic N fixation between rhizobia and legumes is recognised as one of the most efficient forms of biological N fixation (Sprent et al. 2017). The formation of the nodule organ is a sign of the successful establishment of the symbiotic relationship between rhizobia and leguminous plants (Roy et al. 2020). The developmental form and structure of nodules are divided into indeterminate and determinate (Roy et al. 2020; Sprent 2007). Indeterminate nodules are typically formed by leguminous plants in tropical or subtropical regions. They have a cylindrical or branched shape with distinct functional zones, generally the meristematic zone (Z1), infection zone (Z2), interzone (IZ), fixation zone (Z3) and senescence zone (Z4) (Ye et al. 2022). In contrast, leguminous plants in temperate regions often form determinate nodules, with a spherical shape with a uniform internal structure lacking distinct functional zones (Jorgensen et al. 1999). Furthermore, in indeterminate nodules, the infected cells show extensive vacuolation and contain highly differentiated bacteroids responsible for N fixation (Puppo et al. 2004). In contrast, in determinate nodules, the infected cells have less vacuolation and contain less differentiated bacteroids (Jorgensen et al. 1999). *Robinia pseudoacacia* , a resilient tree species with strong adaptability and rapid growth, is an excellent choice for soil restoration and conservation (Deng et al. 2020). Through symbiotic N fixation with rhizobia, *R. pseudoacacia* thrives in arid and saline environments and shows remarkable resistance to heavy metal pollution (Hu et al. 2023; Huo et al. 2022). Consequently, it has emerged as a pioneering species for improving soil conditions, combating desertification and enhancing landscaping projects (Luo et al. 2019; Minucci et al. 2019). Although *R. pseudoacacia* can form indeterminate nodules with rhizobia, the mechanisms underlying nodule development remain poorly understood.

H_2_S is an essential endogenous gaseous signalling molecule that plays a multifaceted role in regulating plant growth and development (Li et al. 2022; Zhang et al. 2021). Accumulating evidence has demonstrated that H_2_S governs seed germination (Zhang et al. 2008; Zhou et al. 2022), root growth (Zhang et al. 2009), stomatal movement (Chen et al. 2020; Pantaleno et al. 2020), flowering (Ma et al. 2021) and autophagy (Aroca et al. 2021). In *Arabidopsis*, H_2_S negatively regulates cytokinin (CTK) synthesis and affects root system architecture (RSA) by persulfidation of CKX2^Cys62^ (Wang et al. 2024). H_2_S also modulates gibberellin (GA) biosynthesis, influencing seed germination and fruit ripening (Li et al. 2021). Furthermore, H_2_S improves drought and salt stress resilience by regulating abscisic acid (ABA) biosynthesis (Chen et al. 2020). Current evidence suggests that H_2_S influences IAA gradients, affecting plant growth and development (Liu et al. 2022). Auxin, a pivotal hormone, regulates numerous essential processes such as embryogenesis, seed development, phyllotaxis, flower and root development (Fiedler and Friml 2023; Leyser 2018). IAA signal transduction relies on the Aux/IAA protein family, which interacts with auxin response factors (ARFs) to regulate downstream gene expression (Tiwari et al. 2001). The *GH3* gene family, encoding acyltransferases that conjugate IAA with amino acids, plays a key role in maintaining IAA homeostasis by forming inactive IAA conjugates (Staswick et al. 2005). This process is critical for regulating IAA activity and maintaining hormonal balance, influencing plant growth and environmental adaptation (Staswick et al. 2005). Our previous studies have demonstrated that H_2_S promotes N fixation in determinate nodules of soybean (Zou et al. 2019), alleviates water stress to maintain the symbiotic relationship between soybean and rhizobia (Lin et al. 2022), enhances N conversion and utilisation in soybean (Zhang et al. 2023), and delays soybean senescence (Liu et al. 2024). However, the role of H_2_S in regulating IAA homeostasis in indeterminate nodule formation processes of woody legume *R. pseudoacacia* remains unclear.

Substantial evidence indicates that H_2_S signalling occurs through persulfidation, a post‐translational modification that converts cysteine residues from ‐SH to ‐SSH, affecting protein interactions, structure and subcellular localisation (Aroca et al. 2017, 2018; Chen et al. 2021; Ma et al. 2023). This modification is a key mechanism by which sulphur compounds regulate plant growth and development, as demonstrated by H_2_S‐mediated persulfidation of Cys103 in ATG18a, which modulates autophagy and influences the size and number of autophagosomes (Aroca et al. 2021). An elevation in H_2_S production induces persulfidation of Cys38 in the NF‐κB p65 subunit, enhancing its binding to ribosomal protein S3 and promoting nuclear translocation to upregulate anti‐apoptotic genes (Sen et al. 2012). H_2_S‐triggered persulfidation disrupts actin polymerisation, impairing root hair growth (Li et al. 2018). In addition, persulfidation modulates key enzymes that maintain reactive oxygen species (ROS) homeostasis and redox balance, including ascorbate peroxidase 1 and GAPDH isoform C1 (GAPC1) (Aroca et al. 2015). In *Arabidopsis*, H_2_S enhances L‐cysteine desulfhydrase 1 (DES1) activity through persulfidation, promoting ABA‐induced stomatal closure and improving drought tolerance (Shen et al. 2020). Our recent findings indicate that decreased H_2_S levels in rhizobia inhibit APX activity via persulfidation of Cys80, disrupting H_2_O_2_ detoxification and promoting accelerated nodule senescence (Liu et al. 2024). But so far, it has not been found that H_2_S and persulfidation affect IAA to regulate indeterminate nodule formation in woody legumes.

IAA concentration is influenced by its biosynthesis and transport and the tightly regulated processes of storage and inactivation, mainly via GH3 family enzymes (Fukui and Hayashi 2018). Thus, modulating GH3.1 activity to regulate IAA levels may represent a novel pathway for regulating plant growth and enhancing symbiotic N fixation. In this study, we found that H_2_S promoted the formation of indeterminate nodules in *R. pseudoacacia* and enhanced N utilisation. Transcriptome profiling revealed that H_2_S upregulates key components of the auxin signalling pathway, notably ARFs and the IAA‐conjugating enzyme GH3.1. We further investigated the role of H_2_S in modulating IAA homeostasis during the nodulation process in *R. pseudoacacia* . Exogenous H_2_S maintained IAA homeostasis under symbiotic conditions and mitigated the inhibitory effects of excessive IAA accumulation on nodule development and nitrogen (N) fixation, underscoring a functional crosstalk between H_2_S and auxin signalling. Mechanistically, we identified Cys304 persulfidation of GH3.1 as a critical molecular target, establishing the basis for H_2_S‐mediated regulation of IAA homeostasis. This study provides crucial insights into H_2_S‐mediated persulfidation and its interaction with IAA signalling pathways in the indeterminate nodule development of the woody legume *R. pseudoacacia* .

## 2 Results

### 2.1 H_2_S Enhances the Nitrogen Uptake and Growth of *R. pseudoacacia*

**FIGURE 1:**
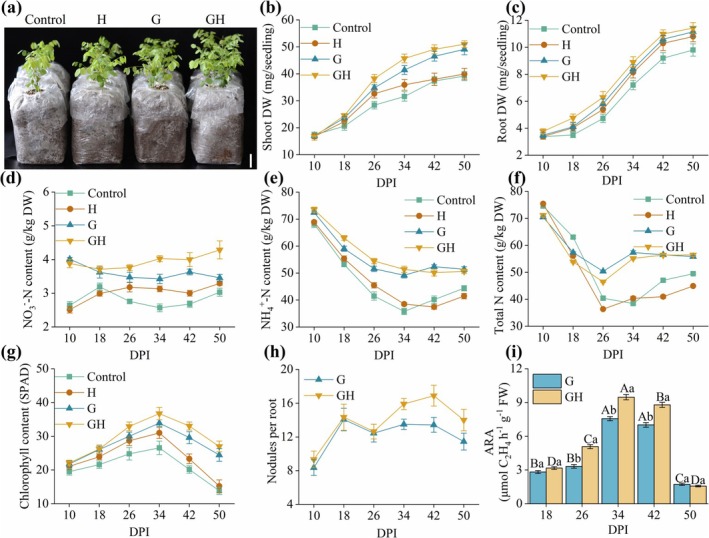
H_2_S enhances nitrogen uptake and plant growth in *Robinia pseudoacacia*. Plant growth and statistics on the physiological indexes and N content of *R. pseudoacacia* . The treatments are as follows: Control (uninoculated), H (uninoculated+100 μM NaHS), G (inoculated with *Mesorhizobium amorphae* GS0123) and GH (inoculated with *M. amorphae* GS0123 and treated with 100 μM NaHS). (a) Growth status of *R. pseudoacacia* seedlings at 26 days post‐inoculation (DPI). Bar, 5 cm. (b) Shoot dry weight of *R. pseudoacacia* . (c) Root dry weight of *R. pseudoacacia* . (d) NO_3_^−^‐N content in the roots of *R. pseudoacacia* . (e) NH_4_^+^‐N content in the roots of *R. pseudoacacia* . (f) Total nitrogen content in the roots of *R. pseudoacacia* . (g) Chlorophyll content across different treatments of *R. pseudoacacia* . (h) Root nodule numbers of *R. pseudoacacia* . (i) Nitrogenase activity assay results of *R. pseudoacacia* . Data are expressed as means ± standard error (SE) from three independent experiments. Uppercase letters indicate significant differences among stages within the same treatment, whereas lowercase letters indicate significant differences among treatments at the same stage (*p* < 0.05).

As depicted in Figure 1a, treatment with 100 μM NaHS significantly enhanced the growth of seedlings in both inoculated and non‐inoculated groups. Notably, under symbiotic conditions, a remarkable increase in the shoot dry weight under NaHS treatment of inoculated plants can be observed (RM GLM, *F* = 5.121, *p* = 0.001) (Figure 1b). Similarly, in non‐inoculated plants, NaHS slightly increased shoot dry weight accumulation at 26 and 34 DPI compared to the Control plants (Figure 1b). The same trend was observed for root dry weight in *R. pseudoacacia* seedlings (RM GLM, *F* = 19.503, *p* = 0.001) (Figure 1c). These results demonstrated that NaHS effectively promoted the growth of *R. pseudoacacia* seedlings within the symbiotic process.

In addition, we determined the effect of exogenous H_2_S on the chlorophyll, total N, NH_4_^+^‐N and NO_3_^−^‐N contents in a *R. pseudoacacia* –rhizobia symbiotic system. Compared to the control group, NaHS treatment increased NO_3_^−^‐N content by 21.8% at 34 DPI. Furthermore, relative to the G group, NaHS led to a 23.8% increase in NO_3_^−^‐N content at 50 DPI, whereas it had no significant effect on NO_3_^−^‐N levels at 10 and 18 DPI (RM GLM, *F* = 3.897, *p* = 0.004) (Figure 1d). Rhizobial inoculation increased NH_4_^+^‐N content in both G and GH groups compared to control plants. In addition, NaHS application slightly enhanced NH_4_^+^‐N levels in H and GH plants from 10 to 34 DPI (RM GLM, *F* = 5.708, *p* = 0.001) (Figure 1e). No significant difference in N content was found among the Control and H treatments at 10 and 18 DPI. However, in terms of total N content, the G and GH groups showed increases of 43.6% and 50%, respectively, compared with control plants at 34 DPI (RM GLM, *F* = 4.951, *p* = 0.001) (Figure 1f). As depicted in Figure 1g, the chlorophyll content was higher in inoculated plants than in non‐inoculated plants from 10 to 50 DPI, especially at 50 DPI; the G and GH groups showed increases of 76.7% and 95%, respectively, relative to the Control plants (RM GLM, *F* = 46.985, *p* = 0.001) (Figure 1g).

To elucidate the role of H_2_S in nodulation, we quantified nodule numbers on *R. pseudoacacia* plants at 10, 18, 26, 34, 42 and 50 DPI to assess the impact of NaHS on nodulation. The application of NaHS dramatically enhanced nodulation from 26 to 50 DPI. The number of nodules in GH groups increased by 25.7% at 42 DPI compared to G groups. However, its effect was not significant during the early stages (10 to 26 DPI) (RM GLM, *F* = 23.045, *p* = 0.001) (Figure 1h). In addition to promoting nodulation, NaHS treatment significantly enhanced the N fixation capacity of nodules. Nitrogenase activity measurement using acetylene reduction analysis (ARA) revealed a significant elevation in nodules from 18 to 42 DPI compared to the G group (Figure 1i). These findings demonstrated that H_2_S promoted indeterminate nodule formation and enhanced the N‐fixing potential of the woody legume *R. pseudoacacia* .

### 2.2 H_2_S Enhances Rhizobial Infection and Modulation of Nodule Gene Expression

**FIGURE 2:**
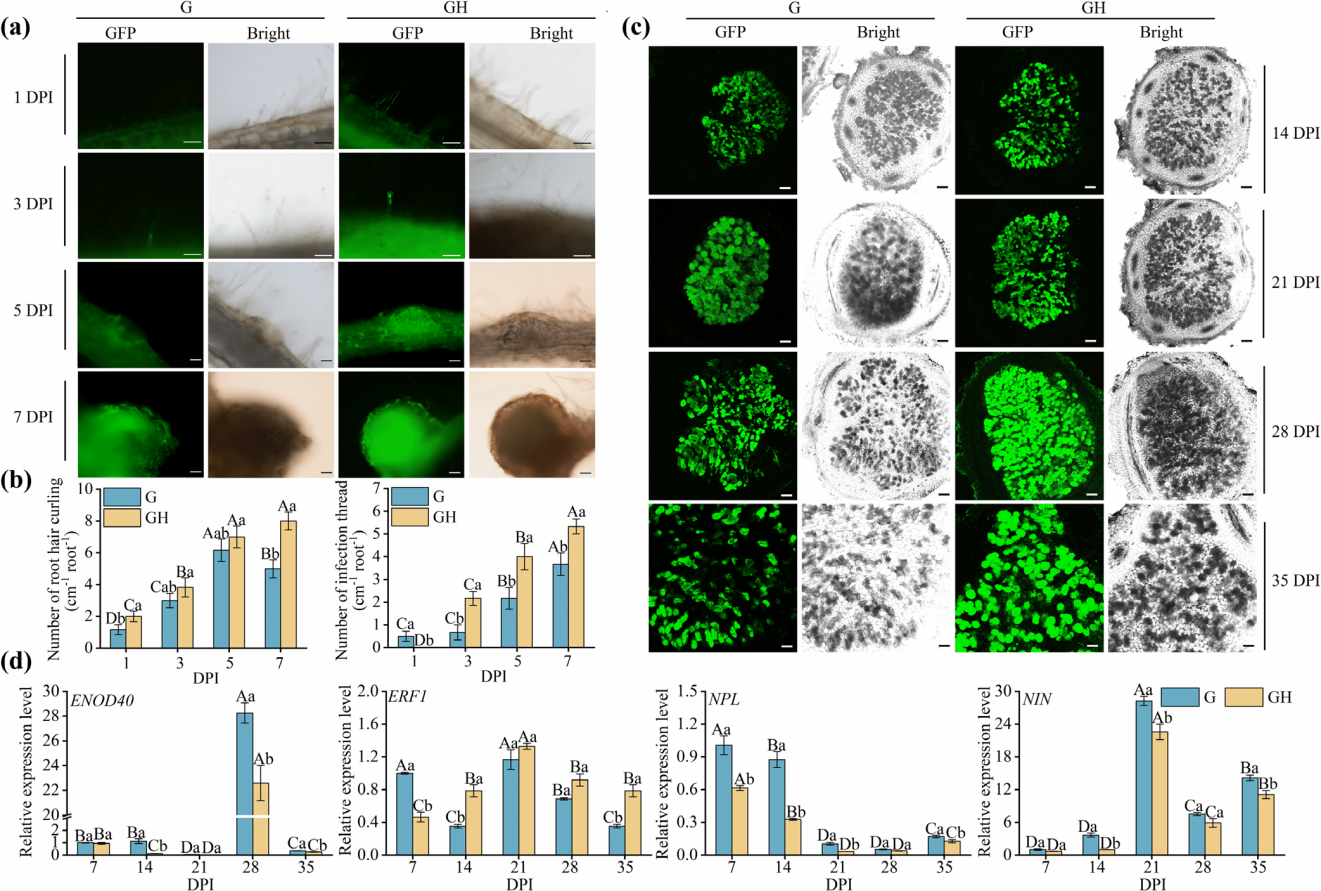
H_2_S‐driven acceleration of rhizobial infection and modulation of nodule gene expression. (a) Plant roots inoculated with the *Mesorhizobium amorphae* GS0123‐GFP were collected at 1, 3, 5 and 7 days post‐inoculation (DPI) for qualitative observation under treatments with or without 100 μM NaHS. (b) Quantification of root hair curling and infection threads (IT). For statistical analysis, the numbers of IT and primordia were assessed from 20 plants (*n* = 20). Scale bars: 100 μm. (c) Colonisation phenotypes of rhizobia. (d) Reverse transcription‐quantitative PCR analysis of *GmENOD40*, *GmERF1*, *GmNPL* and *GmNIN*. Expression levels are relative to G groups and normalised to *ACTIN2* and *GAPDH*. Uppercase letters indicate significant differences among stages within the same treatment, whereas lowercase letters indicate significant differences among treatments at the same stage (*p* < 0.05).

To further elucidate the role of H_2_S during the early stages of symbiosis and nodule formation in *R. pseudoacacia* , we analysed the differences in rhizobial colonisation and nodule primordium formation at different time points (1, 3, 5 and 7 DPI) using fluorescence imaging of roots from the G and GH groups. At 1 DPI, root hairs in the G group exhibited minimal rhizobial attachment, whereas the GH group displayed stronger fluorescence signals in root hairs (Figure 2a). By 3 DPI, infection threads were visible in the GH group, accompanied by a higher level of rhizobial infection of cortical cells compared to the G group (Figure 2a). At 5 DPI, the GH group exhibited a more intense and localised fluorescence signal throughout the cortex, reflecting the ability of rhizobial colonisation. In contrast, the G group showed weaker fluorescence intensity within the nodule primordia, indicating less rhizobial colonisation (Figure 2a). By 7 DPI, both groups had developed visible nodule primordia; however, those in the GH group appeared more organised and exhibited stronger fluorescence, suggesting higher rhizobial density and colonisation efficiency (Figure 2a). Quantitative analysis of root hair curling and infection thread numbers over time (Figure 2b) further confirmed that H_2_S enhanced root responsiveness to rhizobial infection signals.

To further evaluate the role of H_2_S in promoting rhizobial colonisation and nodule development, we conducted fluorescence imaging of nodule sections from 14 to 35 DPI (Figure 2c). At 14 DPI, the GH group displayed slightly stronger fluorescence signals than the G group. From 21 to 28 DPI, fluorescence intensity in the GH group increased progressively, accompanied by a greater number of visibly infected nodule cells and expanded colonisation zones (Figure 2c, Figure S1). By 35 DPI, strong fluorescence was still maintained in the GH group, whereas the G group exhibited a marked reduction in signal intensity (Figure 2c, Figure S1). These observations were further supported by quantitative analysis of fluorescence intensity using ImageJ (Schindelin et al. 2012), which revealed that H_2_S treatment consistently resulted in higher total fluorescence and increased numbers of infected nodule cells across multiple time points (Figure S1). Together, these data suggest that H_2_S facilitated bacteroid colonisation and contributed to the maturation of indeterminate nodules during the later stages of symbiosis.

Nodulation gene expression analysis revealed that H_2_S plays a molecular regulatory role during nodule formation and development. Notably, *ENOD40* expression in nodules at 28 DPI was significantly higher than at 7–21 DPI, and sharply decreased at 35 DPI. The expression of *ERF1* was induced by H_2_S at 14–35 DPI and *NPL* gradually decreased over time following inoculation (Figure 2d). *NIN* expression was strongly upregulated at 21 DPI, and its levels at 28–35 DPI remained significantly higher compared to 7–14 DPI (Figure 2d). In roots, the expression of *ENOD40* and *NPL* was particularly prominent at 3–7 DPI, with a marked increase at 7 DPI upon H_2_S treatment (Figure S2). Additionally, the expression of nucleoporin 133 (NUP133), histidine protein kinase (LHK), calcium/calmodulin‐dependent protein kinase (CCaMK) and the phosphorylation substrate of CCaMK (CYCLOP) was significantly induced by H_2_S at various early stages of symbiosis (Figure S2). The overall regulatory effect of H_2_S on nodulation genes does not follow a continuous enhancing trend. While H_2_S may conservatively inhibit the expression of specific nodule genes in nodules, it significantly elevated the expression levels of *ENOD40*, *NIN*, *NUP133*, *LHK*, *CCaMK* and *CYCLOP* at distinct early stages of root development. Overall, H_2_S facilitates the progression of indeterminate nodule formation by modulating symbiotic gene expression, underscoring its critical role in establishing the symbiotic relationship between *R. pseudoacacia* and rhizobia.

### 2.3 The Regulation of H_2_S on Multiple Stages in Indeterminate Nodule Formation in *R. pseudoacacia*

**FIGURE 3:**
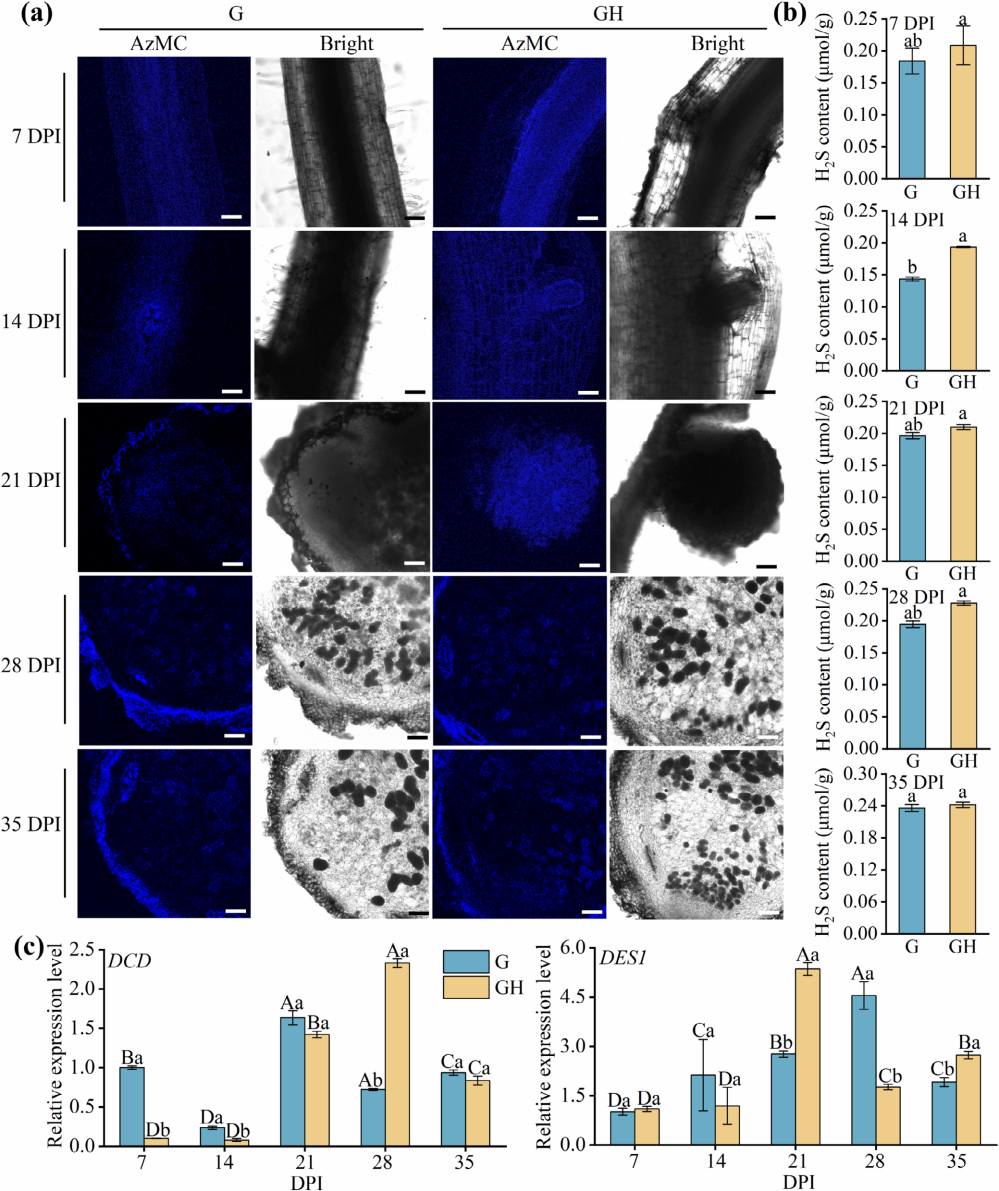
The regulation of H_2_S on multiple stages in indeterminate nodule formation in *Robinia pseudoacacia*. (a) Fluorescence intensity of H_2_S detected by AzMC in roots and nodules were collected at 7, 14, 21, 28 and 35 days post‐inoculation (DPI) in G and GH groups. Bars represent 250 μm. Representative images from 15 nodule sections are shown (5 nodules from different plants per treatment, each with 3 sections). (b) H_2_S content in roots and nodules of *R. pseudoacacia* . (c) Reverse transcription‐quantitative PCR analysis of *DESI* and *DCD* gene expression in nodules. *ACTIN2* and *GAPDH* were employed for the normalisation of plant genes. Data are represented as means ± SD from three independent experiments. Uppercase letters indicate significant differences among stages within the same treatment, whereas lowercase letters indicate significant differences among treatments at the same stage (*p* < 0.05).

The AzMC probe was used to measure H_2_S‐dependent fluorescence intensity in roots and nodules of the G and GH groups at 7, 14, 21, 28 and 35 DPI, allowing assessment of endogenous H_2_S levels throughout the symbiotic process. Our results revealed that the GH group consistently exhibited stronger fluorescence signals than the G group, particularly in the root meristematic regions and nodule primordia (Figure 3a). At 7 DPI, the GH group showed enhanced fluorescence in the roots. By 14 and 21 DPI, fluorescence signals in the GH group were further enhanced by 3.5‐ and 3.39‐fold, respectively (Figure 3a, Figure S3), consistent with the continuous increase in H_2_S content observed during this period (Figure 3b), reflecting the key role of H_2_S in nodule structural development. Although fluorescence signals in both groups showed no significant change in relative intensity at 28 DPI, the GH group maintained higher fluorescence levels at 35 DPI (Figure 3a, Figure S3). This partially contrasts with our direct quantification of H_2_S content (Figure 3b), highlighting the complexity of H_2_S detection. Nonetheless, the above results indicate that NaHS treatment increases H_2_S levels in roots and nodules during nodulation.

In addition, H_2_S significantly upregulated the transcription of the endogenous H_2_S‐synthesising gene *DCD* at 28 DPI. The upregulation of *DES1* was most pronounced at 21 DPI, with a similar effect observed at 35 DPI compared to the G group (Figure 3c). Interestingly, when *DES1* expression was significantly suppressed by H_2_S at 28 DPI, *DCD* expression was markedly increased, likely as a compensatory feedback mechanism to maintain H_2_S homeostasis (Figure 3c), suggesting a coordinated regulatory mechanism of endogenous H_2_S‐synthesising genes during symbiotic nodule formation.

### 2.4 H_2_S Facilitates Bacteroid Colonisation and Functional Zoning of Indeterminate Nodules in *R. pseudoacacia*

**FIGURE 4:**
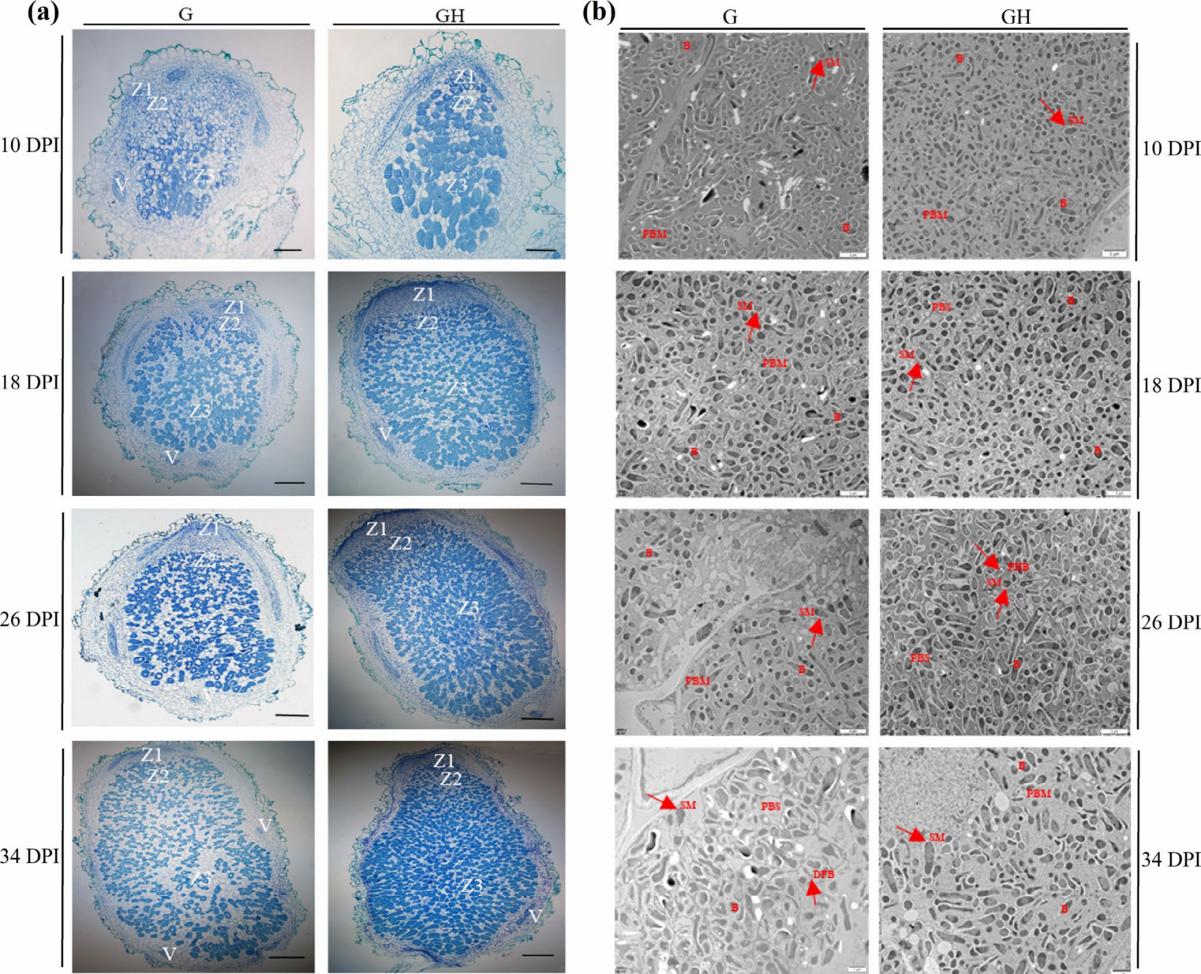
H_2_S enhances bacteroid differentiation and nitrogen fixation zone formation in indeterminate nodules of *Robinia pseudoacacia* . (a) Nodules were stained with toluidine blue at 10, 18, 26 and 34 days post‐inoculation (DPI) (*n* = 3 for each time point). The meristem zone (Z1), infection zone (Z2) and fixation zone (Z3) are indicated. Bars represent 100 μm. (b) Electron micrographs of nodule sections from the G and GH groups at 10, 18, 26 and 34 DPI. In images, Red letters and arrows represent: SM, symbiosome; B, bacteroid; PBS, peribacteroid space. Bars represent 2 μm. Representative images are shown from three biological replicates.

Given the promotive role of H_2_S in early rhizobial infection, we further investigated the subsequent stages of nodule development using light and transmission electron microscopy (TEM). Semithin nodule sections stained with toluidine blue were examined under a light microscope, revealing distinct structural differences between the G and GH groups at multiple time points (10, 18, 26 and 34 DPI) (Figure 4a). In the GH group, the meristematic zone (Z1) was reduced, while the N fixation zone (Z3) was markedly expanded at 10, 26 and 34 DPI, suggesting that H_2_S treatment enhanced rhizobial colonisation compared to the G group (Figure 4a, Figure S4a). Particularly at 10 DPI, a few rhizobia were observed within the IT matrix of the G group's Z2 zone but were scarcely stained (Figure 4a). Notably, H_2_S consistently promoted the expansion of the Z3 zone over time (Figure 4a, Figure S4a), suggesting that H_2_S facilitates sustained rhizobial infection and differentiation of the N fixation region.

TEM analysis further confirmed ultrastructural differences in bacteroids between the G and GH groups. At 10 DPI, the GH group exhibited an increased abundance of small bacteroids (≤ 2 μm in diameter), whereas bacteroid detachment was frequently observed in the G group (Figure 4b, Figure S4b). By 18 DPI, small bacteroids (≤ 1 μm) were more abundant in the GH group, while those in the G group displayed considerable variability in size and morphology. By 26 DPI, bacteroids in the GH group frequently exhibited elongation and a diverse size distribution, with relatively intact symbiosome membranes compared to the G group (Figure 4b, Figure S4b). Notably, at 34 DPI, the GH group preserved relatively intact symbiosome structures and a high abundance of bacteroids. In contrast, the G group occasionally showed signs of early senescence, including a reduced number of bacteroids, particularly those ≤ 2 μm, as well as shrunken bacteroids and disrupted symbiosome membranes (Figure 4b, Figure S4b). Collectively, these ultrastructural observations suggest that H_2_S promotes the differentiation of the N‐fixing zone within nodules and preserves bacteroid structural integrity and division during nodule development.

### 2.5 Transcriptomic Responses of Roots and Nodules to H_2_S in *R. pseudoacacia* –Rhizobia Symbiosis

**FIGURE 5:**
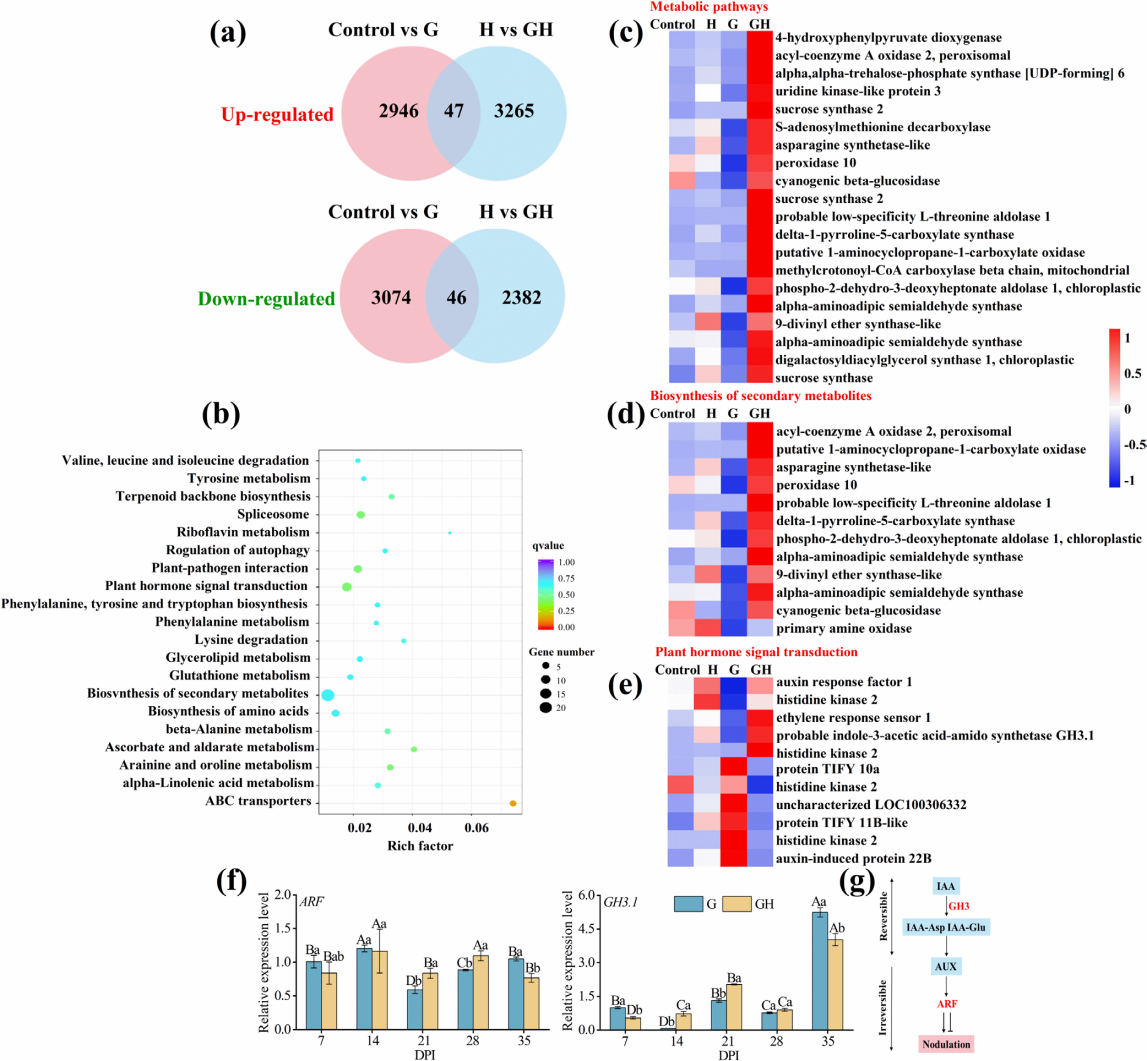
Transcriptomic changes in root responses to H_2_S during *Robinia pseudoacacia* –rhizobia symbiosis. Roots were collected from G and GH groups at 34 days post‐inoculation (DPI). (a) Venn diagram illustrating differentially expressed genes influenced by H_2_S. (b) Enrichment pathways affected by H_2_S. (c) Effects of H_2_S on gene enrichment in metabolic pathways. (d) Effects of H_2_S on gene enrichment in the biosynthesis of secondary metabolites. (e) Effects of H_2_S on gene enrichment in plant hormone signal transduction. (f) Reverse transcription‐quantitative PCR analysis of *ARF* and *GH3.1* gene expression, relative to G group and normalised to *ACTIN2* and *GAPDH*. Data are presented as means ± SD from three independent experiments. (g) Regulatory patterns of IAA, GH3.1 and ARF in the nodulation process. Uppercase letters indicate significant differences among stages within the same treatment, whereas lowercase letters indicate significant differences among treatments at the same stage (*p* < 0.05).

To elucidate the role of H_2_S in regulating the indeterminate nodule development of *R. pseudoacacia* , we conducted transcriptomic analysis on host root and nodule tissues collected at 34 DPI. In the root transcriptome, 2993 genes were identified as upregulated following rhizobial inoculation, along with 3265 additional genes activated by exogenous H_2_S. A subset of 3120 genes was explicitly downregulated in rhizobia‐inoculated samples, while H_2_S treatment caused this downregulation to 2382 unique gene targets. Under both conditions, 46 genes were commonly downregulated (Figure 5a). These differentially expressed genes were predominantly enriched in pathways related to ABC transporters, metabolic processes, secondary metabolite biosynthesis and plant hormone signalling (Figure 5b). The application of H_2_S significantly enhanced the expression of genes associated with host root energy metabolism, including key enzymes such as 4‐hydroxyphenylpyruvate dioxygenase and sucrose synthase, underscoring the importance of H_2_S in tyrosine and sucrose metabolism (Figure 5c). Additionally, H_2_S upregulated genes involved in lipid biosynthesis, such as 9‐divinyl ether synthase‐like (Figure 5d), which are often associated with stress‐related responses in plants (Chehab et al. 2007). Moreover, H_2_S modulated transcriptomic changes and symbiotic gene expression in *R. pseudoacacia* nodules. At 34 DPI, transcriptomic analysis of nodules identified 63 specific genes expressed in rhizobial‐inoculated groups, with an additional 17 genes uniquely expressed upon H_2_S treatment. Notably, both groups shared 6788 common genes (Figure S5a). Gene Ontology analysis revealed significant enrichment of differentially expressed genes related to sulphur metabolism and ABC transporters (Figure S5b). H_2_S inhibited the expression of energy transport‐related ABC transporters and formate dehydrogenase while enhancing sulphate exporter and *HyaD* expression (Figure S5c).

Furthermore, H_2_S induced gene expression in antioxidant mechanisms and hormone signalling. For instance, the peroxisome protein and asparagine synthetase (ASNS) are implicated in ROS detoxification and energy conversion, respectively (Figure 5d, Figure S6a). H_2_S also facilitated the final step of ethylene biosynthesis via 1‐aminocyclopropane‐1‐carboxylate oxidase (ACO) at 7 DPI (Figure S6a). In later stages, H_2_S activated key components of auxin signalling in *R. pseudoacacia* roots and nodules through Auxin Response Factor 1 (ARF1) and GH3.1 at 21 DPI (Figure 5e,f), while simultaneously suppressing the expression level of Histidine Kinase 2 (HK2), *TIFY10* and other auxin‐related genes (Figure 5e, Figure S6c). At 35 DPI, H_2_S similarly suppressed the expression of *ARF* and *GH3.1*, mirroring the pattern observed at 7 DPI (Figure 5f), highlighting its role as a key regulator in the IAA signalling pathway. Based on the regulatory mechanism among IAA, GH3 and ARF (Hayashi et al. 2021), we speculate that H_2_S regulates the expression of *GH3*, thereby affecting the level of IAA and promoting the formation of nodules in *R. pseudoacacia* (Figure 5g).

### 2.6 H_2_S Modulates IAA Homeostasis to Promote Nodule Formation and Nitrogen Fixation

**FIGURE 6:**
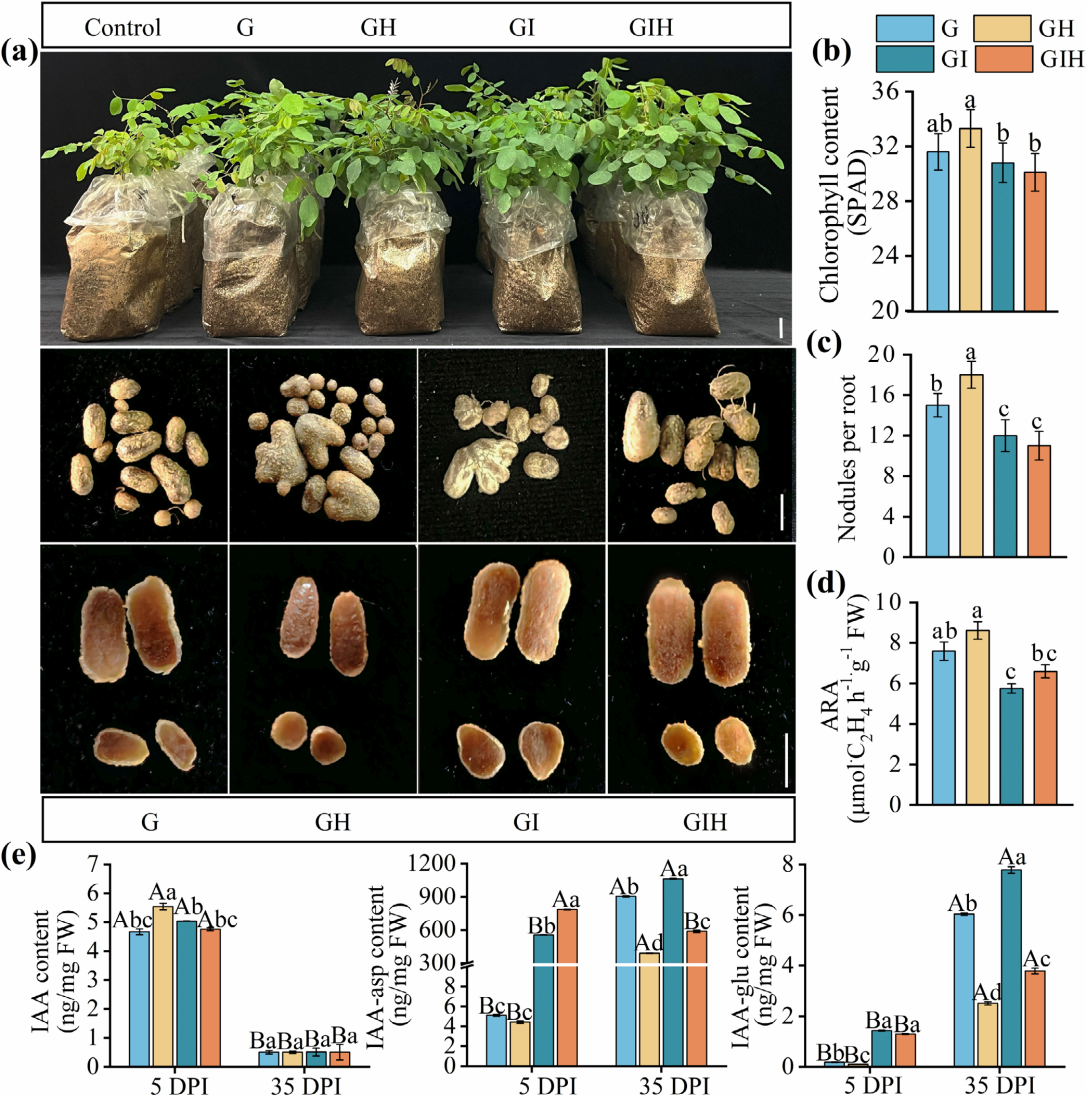
H_2_S‐mediated modulation of IAA homeostasis in nodule formation and nitrogen fixation regulation. Morphological and functional characteristics of control, G, GH, GI ( *Mesorhizobium amorphae* GS0123 + 100 μM IAA) and GIH ( *M. amorphae* GS0123 + 100 μM IAA + 100 μM NaHS) groups. (a) Plant growth phenotype, root nodules and cross‐sectional phenotypes of nodules at 35 days post‐inoculation (DPI). The upper panel depicts the overall growth of the plant (bar = 5 cm), and the lower panel shows the number of nodules per plant (bar = 5 mm). (b) Chlorophyll content. (c) Root nodule number. (d) Nitrogenase activity assay. (e) IAA, IAA‐Asp and IAA‐Glu content in roots at 5 DPI and nodules at 35 DPI. Three independent experiments were conducted. For nodule number, data are means ± standard error (SE) (*n* = 30). For nitrogenase activity, data are means ± SE (*n* = 20). Uppercase letters indicate significant differences among stages within the same treatment, whereas lowercase letters indicate significant differences among treatments at the same stage (*p* < 0.05).

To investigate the interplay between H_2_S and IAA in regulating the symbiotic nodulation process, exogenous H_2_S and IAA treatments were applied, and their effects on plant growth phenotypes and indeterminate nodule development were assessed. As shown in Figure 6a, H_2_S significantly improved the growth status of *R. pseudoacacia* and promoted indeterminate nodule development. Plants in the GH group exhibited darker green leaves, significantly more nodules than other treatment groups, and uniformly whole nodules with dark red cross‐sections, indicative of higher leghaemoglobin content (Figure 6a). Physiological analyses revealed that chlorophyll content in the GH group was significantly higher than in other groups, with particularly pronounced differences compared to the control and GIH groups (Figure 6b). Regarding nodule numbers, the GH group exhibited a significantly higher number of nodules than other groups (Figure 6c), and its nitrogenase activity was also superior to that of different treatment groups. Notably, H_2_S alleviated the inhibitory effect of exogenous IAA on nitrogenase activity in *R. pseudoacacia* (Figure 6d).

To further elucidate the effects of H_2_S on IAA levels in *R. pseudoacacia* , dynamic changes in hormone levels were measured in indeterminate nodules during the early inoculation stage (5 DPI) and N fixation maturity stage (35 DPI). The results showed that indeterminate nodule IAA content was significantly higher at 5 DPI than 35 DPI, particularly in the GH group. Conversely, exogenous IAA reduced free IAA content in indeterminate nodules while significantly increasing conjugated IAA (IAA‐Asp and IAA‐Glu) levels (Figure 6e). Notably, at 35 DPI, H_2_S significantly reduced the levels of IAA‐Asp and IAA‐Glu (Figure 6e), suggesting that H_2_S can effectively reduce the accumulation of conjugated IAA, thereby promoting early symbiotic nodulation in *R. pseudoacacia* .

### 2.7 IAA Modulates H_2_S Distribution and Co‐Regulates Nodulation and Hormonal Signalling Genes

**FIGURE 7:**
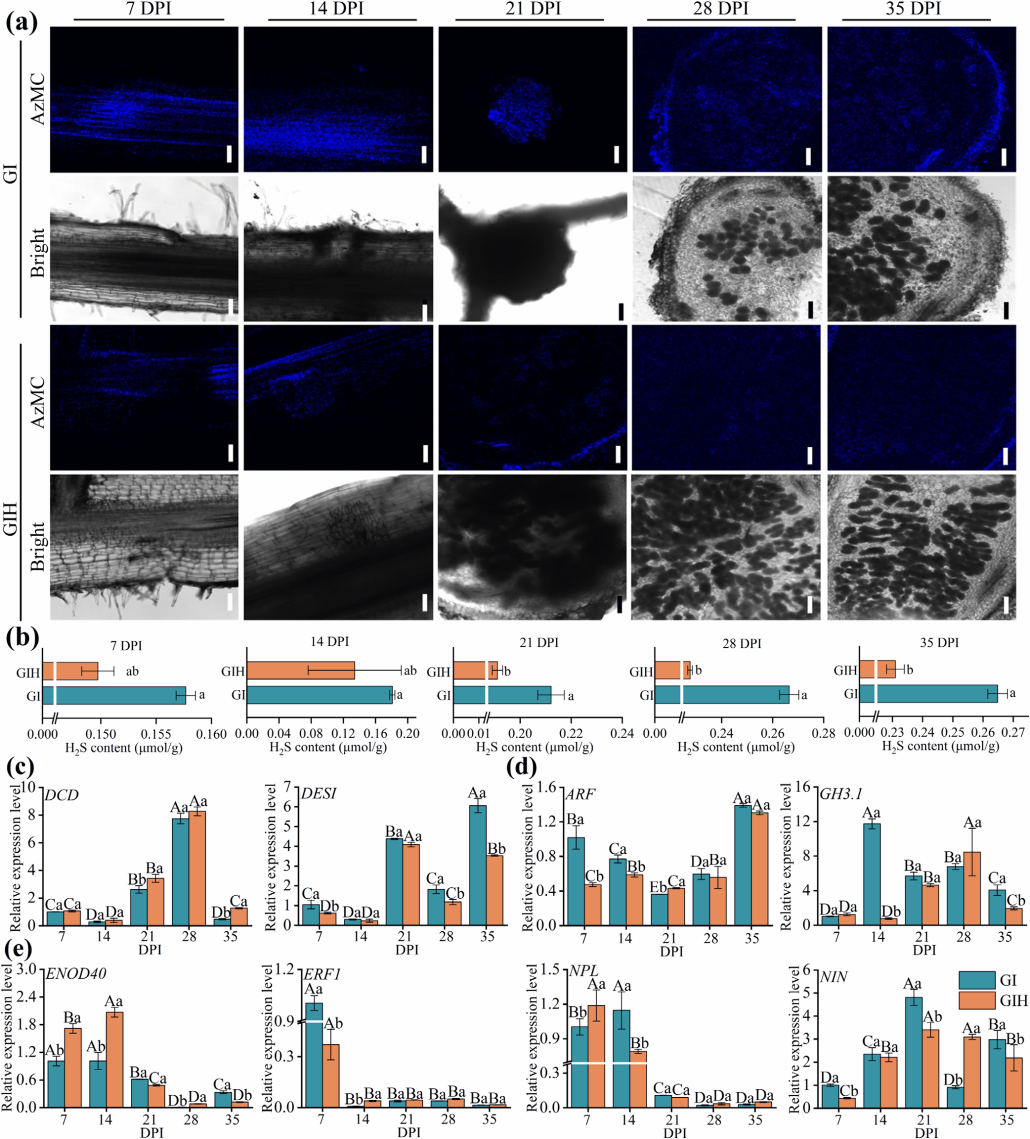
IAA modulates H_2_S distribution and co‐regulates nodulation and hormonal signalling genes. (a) Biochemical characteristics of nodules' microscopic and ultramicroscopic structure inoculated with GI and GIH groups at 7, 14, 21, 28 and 35 days post‐inoculation (DPI). Assays of H_2_S content were performed using the fluorescent probe 7‐azido‐4‐methylcoumarin (AzMC). A total of 15 nodule sections (5 nodules from different plants for each treatment, each with 3 sections) were analysed, and representative images were shown. Bars represent 250 μm. (b) H_2_S content in roots or nodules of *Robinia pseudoacacia* . (c) Reverse transcription‐quantitative PCR analysis of *DESI* and *DCD* expression. (d) Expression of *ARF* and *GH3.1*. (e) Expression of *GmENOD40*, *GmERF1*, *GmNPL* and *GmNIN*; all expression levels are relative to G samples and normalised to *ACTIN2* and *GAPDH*. Uppercase letters indicate significant differences among stages within the same treatment, whereas lowercase letters indicate significant differences among treatments at the same stage (*p* < 0.05).

To elucidate the interplay between H_2_S and IAA during the symbiotic nodulation process, we employed the AzMC probe to examine the temporal–spatial distribution of H_2_S in *R. pseudoacacia* roots and nodules. From the early nodule formation stage (7 DPI) to the N fixation maturity stage (35 DPI), IAA treatment significantly enhanced H_2_S fluorescence signals in roots and nodules (Figure 7a, Figure S7). However, exogenous H_2_S attenuated H_2_S fluorescence signals in roots and nodules of the GIH group (Figure 7a, Figure S7). Similarly, IAA markedly increased H_2_S content in roots and nodules at 7–35 DPI, whereas H_2_S content in the GIH group exhibited a continuous decline compared to the GI group (Figure 7b). The addition of H_2_S induced varying levels of *DCD* expression in *R. pseudoacacia* nodules from 21 to 35 DPI following IAA treatment. In contrast, *DES1* expression was significantly suppressed by H_2_S during the same period, with a notable decrease of approximately 1.5‐fold at 35 DPI (Figure 7c). This indicates that DES1, rather than DCD enzyme, is the primary contributor to endogenous H_2_S production in *R. pseudoacacia* .

Further analysis revealed that IAA significantly induced *ARF* gene expression at 7 DPI (Figure 7d), emphasising its regulatory influence on plant hormone response factors. Additionally, exogenous IAA treatment led to the accumulation of conjugated IAA forms (IAA‐Asp and IAA‐Glu) compared to the G group (Figure 6e), as evidenced by a 12‐fold increase in *GH3.1* expression in the GI group at 14 DPI (Figure 7d). This suggests that IAA promotes the conversion of free IAA to its conjugated forms, thereby reducing free IAA activity (Figure 6e). Notably, H_2_S reduced the IAA‐induced expression of *GH3.1* in *R. pseudoacacia* nodules, observed at 14, 21 and 35 DPI (Figure 7d). Interestingly, IAA and H_2_S treatment significantly upregulated *ENOD40* expression at 7–14 DPI, inhibited *ERF1* expression at 7 DPI, but had no significant effect on its later expression. H_2_S significantly enhanced *NPL* and *NIN* expression at 7 and 28 DPI, respectively. However, *NPL* was transiently suppressed at 14 DPI and maintained low expression levels thereafter (21–35 DPI), while *NIN* was continuously downregulated at 7–21 and 35 DPI (Figure 7e). Based on these findings, H_2_S alleviated the inhibitory effects of excessive exogenous IAA application on nodulation (Figure 6a) by reducing the levels of IAA‐Asp and IAA‐Glu (Figure 6e) and significantly upregulating *ENOD40* expression (Figure 7e). These results suggest that H_2_S responds to IAA levels to modulate its homeostasis and regulate nodulation gene expression, thereby promoting nodule formation in *R. pseudoacacia* .

### 2.8 Persulfidation of Cys304 Diminishes GH3.1 Activity In Vitro

**FIGURE 8:**
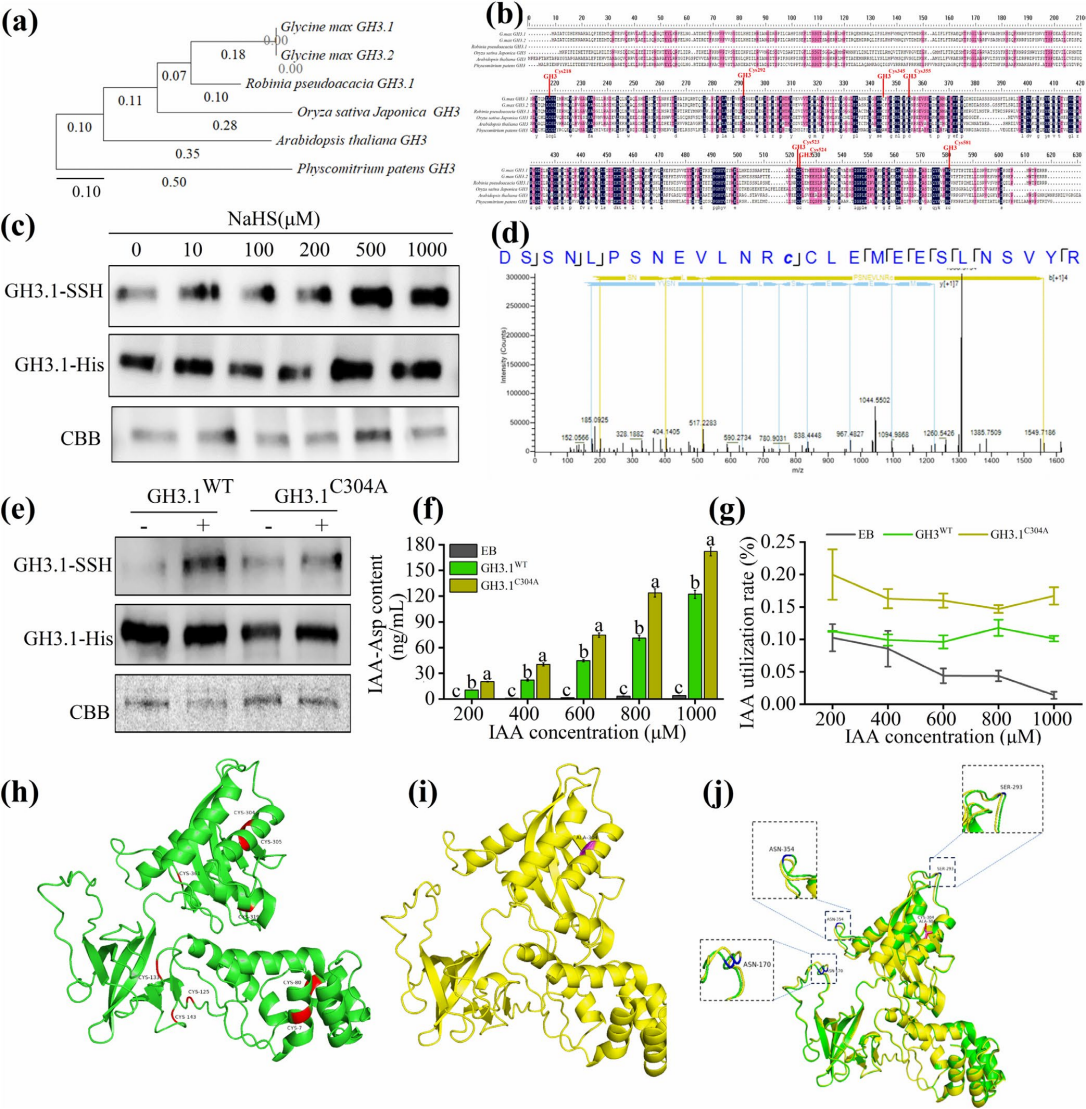
Persulfidation of Cys304 diminishes GH3.1 activity in vitro. (a) Evolutionary tree of GH3s protein family. (b) Sequence alignment of GH3s protein family. The red marker indicates the sites corresponding to Gretchen Hagen 3 (GH3) persulfidated cysteine in different proteins. (c) Analysis of liquid chromatography–tandem mass spectrometry (LC–MS/MS) concerning GH3.1 persulphide modifications induced by H_2_S. Persulfidation of GH3.1 was detected using the modified biotin‐switch method (MBSM). Equal amounts of protein were incubated for 30 min and detected by immunoblotting with an anti‐biotin antibody. An anti‐His antibody served as the control. Coomassie Brilliant Blue (CBB) staining was used as a loading control. (d) Fully annotated MS/MS spectra of IAM‐derivatized GH3.1 peptides containing persulfidated cysteines. (e) In vitro persulfidation analysis of GH3.1 and mutant recombinant proteins using the tag‐switch method; persulfidated proteins were detected with an anti‐biotin antibody. (f) Effects of persulfidation on GH3.1 activity (in Elution Buffer [EB] wild‐type and C304A mutants), treated with either control or 200–1000 μM NaHS. (g) GH3.1 (in EB and wild‐type and C304A mutants) has the ability to bind aspartate (Asp). Values are means ± SE from at least three biological replicates. Bars with different letters indicate significant differences (*p* < 0.05). (h) Structural diagram of GH3.1^WT^ (green). (i) Structural diagram of GH3.1^C304A^ (yellow). (j) Structural details comparing GH3.1 wild type and GH3.1^C304A^.

To clarify the evolutionary degree and conserved structure of GH3 among different species, we conducted a sequence alignment of the GH3 protein family and constructed an evolutionary tree (Figure 8a). Sequence alignment analysis indicated that the GH3 family members showed conservation in their sequences (Figure 8b). The GH3.1 of the woody legume *R. pseudoacacia* was closely related to GH3.1 and GH3.2 in the crop soybean, sharing seven cysteine residues available for persulfidation, namely Cys218, Cys292, Cys345, Cys355, Cys523, Cys524 and Cys581 in soybean and Cys7, Cys80, Cys133, Cys143, Cys304, Cys305 and Cys361 in *R. pseudoacacia* (Figure 8a,b). The cysteine persulfidation sites of Cys7, Cys80, Cys304, Cys305 and Cys361 in *R. pseudoacacia* were conserved in GH3.1. To investigate the molecular mechanism by which H_2_S regulates IAA homeostasis, we employed a modified biotin‐switch method (MBSM) to detect the persulfidation (SSH) levels of the GH3.1 protein under varying NaHS concentrations. The results demonstrated a concentration‐dependent increase in GH3.1 persulfidation levels with rising NaHS concentrations (10–1000 μM), confirming the reliability of the experiment through GH3.1‐His and Coomassie Brilliant Blue (CBB) staining (Figure 8c). Moreover, we identified Cys304 as the specific persulfidation site on GH3.1 by liquid chromatography‐tandem mass spectrometry (LC–MS/MS) (Figure 8d). These findings indicate that H_2_S modifies the GH3.1 protein in a concentration‐dependent manner, highlighting its potential role in regulating GH3.1 enzymatic activity.

We mutated Cys304 to alanine (Ala) and assessed the response of the mutant protein to H_2_S. Compared to the wild‐type GH3.1 (GH3.1^WT^), the mutant protein GH3.1 (GH3.1^C304A^) markedly abolished H_2_S‐induced persulfidation (Figure 8e). To examine the impact of persulfidation on GH3.1 function, we quantified its enzymatic activity by monitoring the conversion of free IAA into conjugated forms under varying H_2_S concentrations. As shown in Figure 8f, compared to the Elution Buffer (EB) group, the content of IAA‐Asp produced by GH3.1^WT^ significantly increased with rising H_2_S concentrations. Similarly, the utilisation rate of IAA in the GH3.1^C304A^ group was significantly higher than that in the GH3.1^WT^ and EB groups (RM GLM, *F* = 5.750, *p* = 0.001) (Figure 8g), suggesting that H_2_S inhibits GH3.1 activity by promoting disulphide bond formation through persulfidation of the Cys304 residue.

Moreover, we analysed the protein structure of GH3.1 using AlphaFold3, highlighting key Cys potentially modified by H_2_S near the enzyme's active centre (Figure 8h). NaHS induced conformational changes in the local loop region of GH3.1, with notable alterations observed in loops containing ASN170, ASN354 and SER293 (Figure 8j). The structures of GH3.1^WT^ and GH3.1^C304A^, represented by green and yellow models, respectively, revealed the classic α/β fold structure of GH3.1 (Figure 8h,i). However, structural alignment of GH3.1^WT^ and GH3.1^C304A^ revealed that mutation at the Cys304 site caused displacement of several loops in the protein structure (Figure 8j). These suggest that H_2_S‐mediated persulfidation at Cys304 may regulate GH3.1 enzymatic activity by inducing conformational changes in the protein structure.

## 3 Discussion

### 3.1 H_2_S Regulates Indeterminate Nodule Formation and Nitrogen Fixation in *R. pseudoacacia*

Establishing plant–microbial symbiosis involves a complex regulatory network where signalling molecules are indispensable (Andrio et al. 2013; Fukuto et al. 2012; Oldroyd 2013). Nitric oxide (NO) production within nodules is crucial for establishing symbiotic associations, as demonstrated in *Medicago truncatula* and rhizobia (Baudouin et al. 2006; del Giudice et al. 2011; Hérouart et al. 2002). NO production occurs during the early infection phase of nodule formation (Nagata et al. 2008), and may synergistically interact with ROS to regulate the initial stages of nodule development (Damiani et al. 2016). Subsequent collaboration between NO and auxin modulates the indeterminate nodule formation in *M. truncatula* (Leach et al. 2010), and impacts nitrogenase activity during N fixation (Cam et al. 2012; Damiani et al. 2016). Our previous studies demonstrated that exogenous H_2_S, delivered via sodium hydrosulphide (NaHS), promotes the development and formation of determinate nodules in soybean while enhancing nitrogenase activity (Zou et al. 2019). Moreover, during N deficiency‐induced senescence, H_2_S and rhizobia expedite N assimilation and remobilisation by regulating senescence‐associated genes, thereby promoting soybean growth (Zhang et al. 2020). Under water stress, H_2_S and rhizobia enhance antioxidant capacity, mitigating oxidative damage in soybeans (Lin et al. 2022). However, the effects of H_2_S on indeterminate nodule formation and N fixation in woody legume *R. pseudoacacia* remain unclear.

Our findings indicate that exogenous H_2_S effectively mitigates N deficiency in *R. pseudoacacia* (Figure 1a) and boosts plant biomass (Figure 1b,c), consistent with previous research (Lin et al. 2022; Zhang et al. 2020; Zou et al. 2019). Besides, H_2_S and rhizobia jointly improved nitrate utilisation efficiency and ammonium absorption capacity (Figure 1d,e), leading to a marked increase in total N content during 26–50 DPI (Figure 1f). This aligns with our findings that H_2_S increased chlorophyll content, as indicated by SPAD measurements (Figure 1g), which are consistent with the report by Rizwan et al. (2019), who demonstrated that H_2_S alleviates N starvation by promoting chlorophyll synthesis and photosynthesis. Furthermore, H_2_S significantly increased nitrogenase activity in indeterminate nodules during 26–42 DPI (Figure 1i). This is consistent with previous findings that host‐derived regulatory signals facilitate terminal differentiation of rhizobial symbionts in indeterminate nodules, which is critical for maintaining N fixation (Gao et al. 2023).

Notably, Pereyra et al. (2015) demonstrated that the effects of H_2_S on N metabolism are amplified under drought stress, suggesting that environmental factors may influence its regulatory impacts. Regarding indeterminate nodule development, H_2_S significantly accelerated the formation and development of indeterminate nodules by promoting key processes, including root hair curling, infection thread formation and rhizobial colonisation (Figure 2a–c). This aligns with findings from Zou et al. (2019), who reported that H_2_S facilitates infection thread formation and rhizobial colonisation in determinate nodules. While H_2_S had limited influence on *NUP85* and *LIN‐1* (Cerberus) during the early symbiotic stages, it notably enhanced the transcription of critical symbiotic regulators, such as *ENOD40*, *SYMRK*, *NUP133*, *LHK*, *CCaMK* and *NIN* at specific time points, thereby facilitating nodule initiation and development (Figure S2). Similar regulatory patterns have been observed in determinate nodules (Zou et al. 2020). It is noteworthy that we also observed a reduction by H_2_S in *ENOD40*, *ERF1*, *NPL* and *NIN* expression in the nodules between 7 and 35 DPI. This regulatory pattern may help sustain the development of indeterminate nodules in *R. pseudoacacia* .

Additionally, we found that H_2_S accumulated to higher levels in *R. pseudoacacia* within GH roots and nodules (Figure 3a,b). Interestingly, we observed a time‐dependent regulation of H_2_S synthase genes, with *DCD* negatively regulated by H_2_S during early inoculation (7–14 DPI) and *DES1* peaking at 21 DPI, highlighting the temporal specificity of H_2_S‐mediated regulation in *R. pseudoacacia* (Figure 3c). These findings are consistent with the role of H_2_S in facilitating both early bacteroid colonisation and later nodule cell differentiation during nodulation (Figure 4a,b). Our previous studies also demonstrated that endogenous H_2_S biosynthesis is essential for maintaining antioxidant capacity in determinate nodules (Zou et al. 2020). These suggest that exogenous H_2_S promotes nodule development by synergistically modulating H_2_S biosynthesis in the host plant.

### 3.2 H_2_S Modulates Transcriptomic Changes and Hormonal Pathways in *R. pseudoacacia* During Symbiotic Nitrogen Fixation

Transcriptomic analysis revealed that H_2_S significantly impacts energy metabolism by upregulating genes involved in sugar metabolism, mainly sucrose and starch (Figure 5c). Sucrose is essential for respiration and provides energy for plant growth, participating in various metabolic pathways (Ruan et al. 2010). Upon translocation to nodules, sucrose undergoes glycolysis to generate phosphoenolpyruvate (PEP), which is subsequently converted to malic acid and pyruvate—key carbon skeletons required for N fixing reactions (Ke et al. 2022). Research indicates that 16% of carbohydrates produced by soybean photosynthesis are utilised by nodules, with a carbohydrate requirement of 15–20 g per gram of fixed N (Stitt and Krapp 2002). This highlights the crucial role of starch and sucrose metabolism in N fixation efficiency. In addition to carbohydrate metabolism, H_2_S promotes the expression of the S‐adenosylmethionine decarboxylase (*SAMDC*) gene (Figure S6a), significantly upregulated at 21 DPI. SAMDC is a key enzyme in polyamine biosynthesis, which is essential for plant growth and development by regulating polyamine levels (Hu et al. 2005). H_2_S also upregulates the *ASNS* gene (Figure S6a), which encodes asparagine synthetase, thereby enhancing N recycling and assimilation (Deng et al. 2022). Plant hormones also play a central role in regulating nodulation (Tu et al. 2024), and our results suggest that H_2_S affects several hormone‐related pathways. Notably, H_2_S treatment markedly increased the expression of *AOC4*, which encodes allene oxide cyclase involved in jasmonic acid (JA) biosynthesis (Figure S6a), indicating potential modulation of JA signalling (Stenzel et al. 2012). Furthermore, *ARF* gene expression was enhanced, particularly at 35 DPI (Figure 5f). *ARFs* are transcriptional regulators that confer specificity to auxin signalling by targeting downstream genes (Li et al. 2016), suggesting that H_2_S contributes to auxin‐mediated developmental regulation during nodulation in *R. pseudoacacia* .

To validate the effect of H_2_S on the expression of nodulation‐related genes during the symbiotic interaction between *R. pseudoacacia* and rhizobia, we focused on eight symbiotic genes involved in rhizobial infection and nodule development: *SYMRK*, *CCaMK*, *Castor*, *CYCLOP*, *NUP85, NUP133*, *Cerberus* and *LHK* (Figures S2 and S8). Although H_2_S did not significantly induce *SYMRK* expression, it markedly upregulated *CYCLOP* under rhizobial challenge, indicating its responsiveness to H_2_S. *CYCLOP* forms an ancient, preassembled signal transduction complex with *CCaMK* that is specifically required for infection (Yano et al. 2008). Moreover, H_2_S enhanced the expression of key nodulation and N fixation genes, including *nifH*, *nodC*, *Zheng44*, *Zheng45*, *Zheng190* and *Zheng273* (Figure S9). Rhizobial nodulation genes are essential for forming effective nodules and maintaining high N‐fixing capacity (Aguilar et al. 2022; Dénarié et al. 1996). These findings improve our understanding of the molecular basis of symbiosis between woody legumes and rhizobia, and clarify the role of H_2_S in regulating N fixation.

### 3.3 H_2_S and IAA Co‐Regulation in Indeterminate Nodule Formation and Nitrogen Fixation in *R. pseudoacacia*

*GH3* family genes regulate active indole‐3‐acetic acid (IAA) levels by catalysing its conjugation with amino acids, forming inactive IAA‐Asp and IAA‐Glu and thus reducing free IAA activity (Staswick et al. 2005). In this study, H_2_S reduced IAA‐Asp and IAA‐Glu levels, maintaining free IAA levels and promoting indeterminate nodule formation and development (Figure 6e), consistent with reports highlighting the importance of IAA gradients in regulating root hair development and organogenesis (Grieneisen et al. 2007; Mähönen et al. 2014). The GIH group exhibited significantly increased chlorophyll content (Figure 6b), likely due to the synergistic effects of H_2_S on IAA signal transduction. Previous studies have shown that H_2_S promotes plant growth by modulating IAA gradients (Fang et al. 2014; Zhang et al. 2009). Additionally, H_2_S alleviated the inhibitory effects of exogenous IAA on nitrogenase activity in *R. pseudoacacia* (Figure 6d), likely through regulation of IAA metabolism. This is consistent with previous findings that mutations in GH3.2 significantly affect plant growth and disease resistance (González‐Lamothe et al. 2012). In our study, H_2_S suppressed *GH3.1* gene expression at 14 DPI (Figure 7d), reducing conjugated IAA levels. These findings further support the important role of H_2_S in regulating IAA metabolism during symbiotic nodulation between woody legumes and rhizobia.

IAA significantly increased H_2_S fluorescence signals and content in *R. pseudoacacia* roots and indeterminate nodules (Figure 7a,b). Our results highlight the crucial role of H_2_S in regulating nodulation via modulation of *DES1* expression and IAA signalling. Although H_2_S induced variable *DCD* expression, it markedly suppressed *DES1*, suggesting that DES1 is the primary enzyme responsible for H_2_S production in *R. pseudoacacia* nodules (Figure 7c), consistent with previous studies (Li et al. 2022; Shen et al. 2020). While IAA treatment enhanced H_2_S distribution and *DES1* expression, it inhibited nodule formation (Figures 6a–c and 7c), aligning with reports of IAA's negative impact on nodulation (Alemneh et al. 2020). However, H_2_S alleviated the inhibitory effects of IAA, suggesting a potential role of H_2_S in modulating IAA signalling during symbiosis. IAA also induced *ARF* gene expression at 7 DPI (Figure 7d), which directly regulates the expression of auxin‐responsive genes (Cho et al. 2024), highlighting its role in early symbiotic signalling. Notably, IAA significantly upregulated *GH3.1* expression at 14 DPI (Figure 7d), potentially resulting in the accumulation of conjugated IAA forms, which inhibited indeterminate nodule formation. This aligns with previous findings that IAA induces *GH3* gene expression and regulates IAA homeostasis through a negative feedback mechanism (Ke et al. 2024).

In our study, the exogenous IAA induced overactivation of GH3.1 and inhibited indeterminate nodule development (Figure 7a–d). Therefore, at the early stage of 14 DPI, the suppression of *GH3.1* by H_2_S suggests that H_2_S modulates IAA metabolism, promoting auxin homeostasis essential for nodule formation. Similarly, the carotenoid‐derived metabolite anchorene regulates auxin homeostasis, primarily by repressing GH3‐mediated IAA conjugation and inactivation pathways (Ke et al. 2024). We also confirmed that exogenous IAA induces the production of H_2_S in *R. pseudoacacia* , yet it did not alleviate the inhibitory effect of IAA on nodule formation. In contrast, exogenous H_2_S significantly enhanced nodulation in the GIH group (Figure 6a–d). Our findings imply that H_2_S may influence IAA signalling upstream, helping maintain active IAA homeostasis. This hypothesis is consistent with our findings on the content of IAA and its amino acid conjugates, showing that H_2_S significantly modulates IAA levels during both early symbiotic stages and N fixation senescence (Figure 6e), consistent with Rekhter et al. (2019), who demonstrated a key role of H_2_S in plant hormone signalling. Moreover, the upregulation of *ENOD40* and the downregulation of *ERF1* expression following combined treatment with conjugated IAA and H_2_S suggest that coordinated regulation of early symbiotic signalling by these molecules. This finding aligns with previous findings on H_2_S‐mediated regulation of nodulation‐related genes in soybean symbiosis (Zou et al. 2020, 2019). Consequently, these results indicate that H_2_S regulates IAA metabolism and signalling via modulation of *GH3.1* expression, thereby promoting indeterminate nodule formation and enhancing growth in woody legumes.

### 3.4 H_2_S‐Mediated Persulfidation of GH3.1 Protein and Its Molecular Mechanism

As a critical gaseous signalling molecule, H_2_S regulates plant growth and stress responses primarily through the persulfidation of cysteine residues in target proteins (Aroca et al. 2018; Gotor et al. 2019). Persulfidation, as a reversible post‐translational modification, converts thiol groups (‐SH) into persulphide groups (‐SSH), modulating protein activity, stability and intermolecular interactions (Zivanovic et al. 2019). In this study, we investigated the molecular mechanism by which H_2_S regulates GH3.1 activity to influence IAA homeostasis. Our results showed that the persulfidation level of GH3.1 protein increased significantly with rising concentrations of NaHS (10–1000 μM), exhibiting a dose‐dependent trend (Figure 8c).

Furthermore, LC–MS/MS analysis identified Cys304 as the specific persulfidation site of GH3.1 (Figure 8c,d). Site‐directed mutagenesis further confirmed this result, as replacing Cys304 with alanine (C304A) markedly reduced GH3.1 persulfidation, verifying Cys304 as the target site of H_2_S‐mediated modification (Figure 8e). This regulatory mechanism is consistent with previous reports in *Arabidopsis*, where H_2_S enhances DES1 activity through persulfidation of Cys44 and Cys205, facilitating ABA‐induced stomatal closure (Shen et al. 2020). Similarly, the persulfidation of RBOHD promotes ROS production in *Arabidopsis*, regulating ABA signalling pathways (Scuffi et al. 2018). Additionally, H_2_S‐mediated persulfidation contributes to the maintenance of ion homeostasis under salt stress by modulating key ion transporters (Ma et al. 2023). Our study further revealed that H_2_S‐induced persulfidation of GH3.1 significantly reduced its enzymatic activity, while the activity of the GH3.1^C304A^ was increased considerably. Notably, the inhibition of GH3.1 activity was dose‐dependent on NaHS treatment (Figure 8f,g). This finding aligns with previous studies showing that H_2_S protects antioxidant enzymes in *Arabidopsis* (Zivanovic et al. 2019) and soybean (Liu et al. 2024) from excessive oxidative damage through persulfidation, maintaining functional stability. Collectively, these studies highlight the widespread role of H_2_S in regulating target protein function through site‐specific persulfidation.

To further investigate the effects of H_2_S on GH3.1 function, we used AlphaFold3 to predict the protein structure of GH3.1. The results showed that persulfidation at the Cys304 residue induced significant conformational changes in the local loop regions of GH3.1, particularly in areas containing ASN170, ASN354 and SER293 (Figure 8j). These loop regions are near the enzyme's active centre and may reduce the catalytic efficiency of GH3.1 by altering the spatial structure of its substrate‐binding sites. Similarly, H_2_S modifies key residues in *Arabidopsis* ATG18a through persulfidation, altering its conformation to regulate autophagy (Aroca et al. 2021; Laureano‐Marín et al. 2020). Likewise, H_2_S has been shown to enhance CTK signalling‐related protein activity through persulfidation, promoting root growth and lateral root formation (Wang et al. 2024). We propose that H_2_S reduces the catalytic activity of GH3.1 by persulfidating its Cys304 site and altering its local conformation (Figure 8i,j). This study demonstrates that H_2_S maintains appropriate IAA levels by reducing GH3.1 activity through persulfidation, thereby influencing indeterminate nodule development in *R. pseudoacacia* .

**FIGURE 9:**
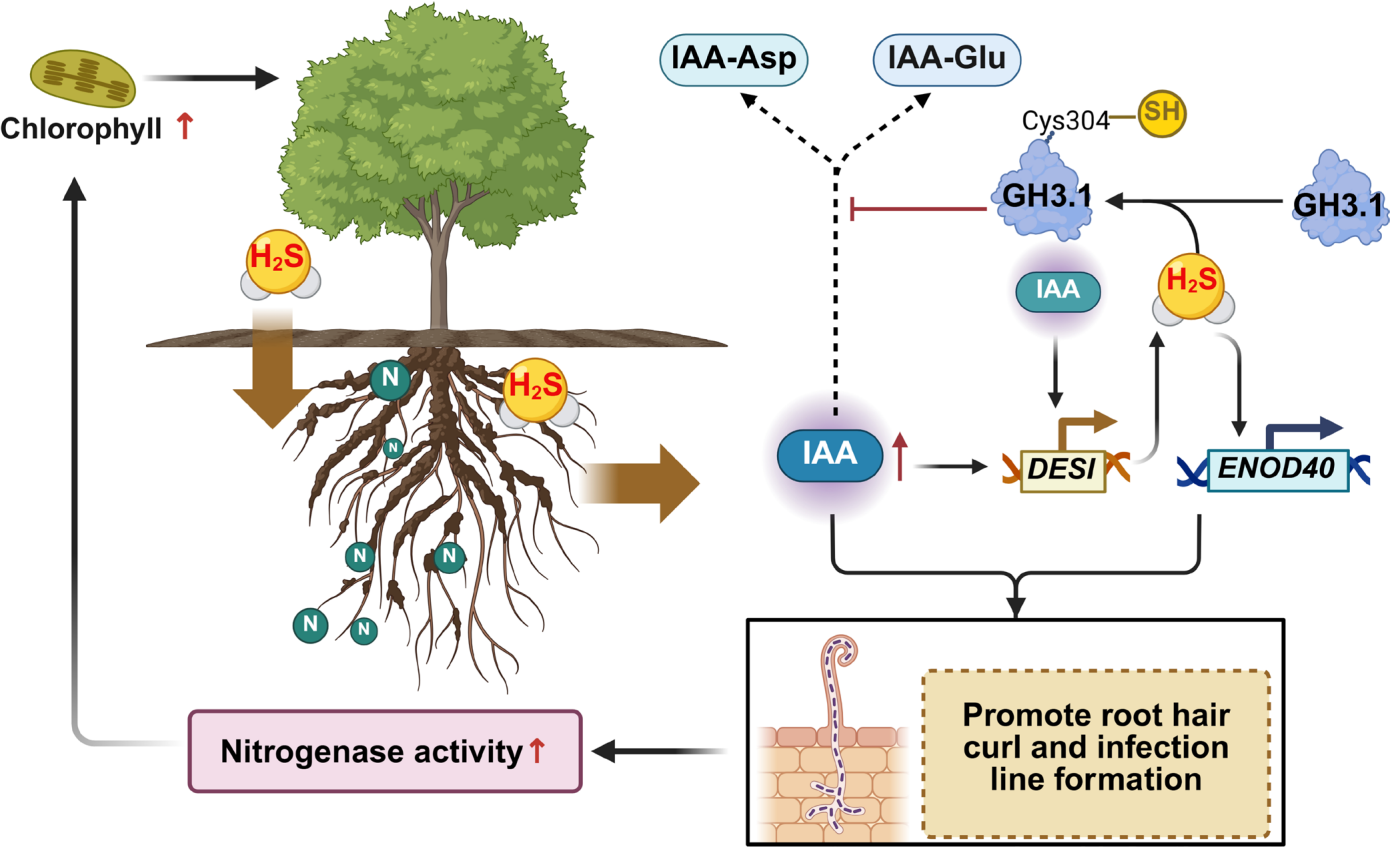
H_2_S regulates GH3 activity to maintain IAA levels and promote nodule formation in *Robinia pseudoacacia* . The model demonstrates that H_2_S persulfidates GH3.1 at Cys304, inhibiting its activity and reducing IAA conjugation (IAA‐Asp/Glu), thereby preserving free IAA levels. This stabilised IAA pool, in synergy with H_2_S, upregulates *DES1* to amplify endogenous H_2_S production, forming a positive feedback loop. Concurrently, the H_2_S‐IAA module enhances *ENOD40* expression, promoting root hair curling and infection thread formation for rhizobial colonisation. These coordinated mechanisms sustain nodule organogenesis, elevate nitrogenase activity and enhance chlorophyll biosynthesis, ultimately improving photosynthetic efficiency and plant growth.

In summary, our study elucidates a mechanistic model (Figure 9) wherein H_2_S modulates IAA homeostasis to facilitate indeterminate nodule development in the woody legume *R. pseudoacacia* . Specifically, H_2_S mediates persulfidation of the Cys304 residue in GH3.1, inducing conformational changes in its loop structure and attenuating its enzymatic activity. This post‐translational modification suppresses the conjugation of free IAA to inactive forms (IAA‐Asp and IAA‐Glu), thereby maintaining optimal free IAA levels. The stabilised IAA in coordination with H_2_S activates a positive feedback loop by upregulating *DES1* expression, thereby enhancing endogenous H_2_S production. The co‐accumulation of IAA and H_2_S enhances *ENOD40* transcription in roots, thereby facilitating root hair curling, infection thread and indeterminate nodule formation. Consequently, these processes lead to increased nitrogenase activity and enhanced chlorophyll biosynthesis. Together, these effects improve plant physiological performance and contribute to the vigorous growth of *R. pseudoacacia* . Our findings advance understanding of the mechanism by which H_2_S modulates auxin homeostasis through persulfidation in indeterminate nodulation in woody legumes, revealing a regulatory pathway that facilitates nodule development and symbiotic N fixation.

## 4 Experimental Procedures

### 4.1 Plant Growth and H_2_S Treatment

The uniform and whole *R. pseudoacacia* seeds were treated with 98% H_2_SO_4_ for 8–10 min, followed by 95% ethanol treatment for 1 min and sterilisation with 50% sodium hypochlorite for 10 min. The seeds were then washed with sterile water for six times (Wang et al. 2019). Surface‐sterilised seeds were cultivated at 28°C in the dark for 48 h. Six hundred millilitres of a 1:1 (vol/vol) mixture of vermiculite and perlite was watered with 350 mL of N‐free Fahraeus nutrient medium (100 mg/L CaCl_2_, 100 mg/L KH_2_PO_4_, 5 mg/L ferric citrate, 150 mg/L NaH_2_PO_4_, 240 mg/L MgSO_4_, 2.86 mg/L H_3_BO_3_, 2.03 mg/L MnSO_4_·4H_2_O, 0.22 mg/L ZnSO_4_·7H_2_O, 0.06 mg/L Na_2_MoO_4_·2H_2_O and 0.08 mg/L CuSO_4_·5H_2_O) and sterilised in a polypropylene planting bag. Germinated seeds were transferred to the growth medium (three seedlings per bag). Plants were grown in a controlled environment chamber under a 16 h light/8 h dark photoperiod, 80% relative humidity, 27°C and photosynthetically active radiation (PAR) of 280 μM m^−2^ s^−1^.

The initial phase of the experiment involved 10‐day‐old *R. pseudoacacia* seedlings were divided into four groups: the first group was treated as the uninoculated group (Control), the second group was treated with 100 μM NaHS (H), the third group was inoculated with *Mesorhizobium amorphae* CCNWGS0123 (G), and the fourth group was inoculated with *M. amorphae* CCNWGS0123 and treated with NaHS (GH). In addition, the experimental treatments in the latter part included five groups: the first group served as the uninoculated group (Control), the second group was inoculated with *M. amorphae* CCNWGS0123 (G), the third group was inoculated with *M. amorphae* CCNWGS0123 and treated with NaHS (GH), the fourth group was inoculated with *M. amorphae* CCNWGS0123 and treated with 100 μM IAA (GI), and the fifth group was inoculated with *M. amorphae* CCNWGS0123 and treated with IAA and NaHS (GIH). All G and GH group seedlings were inoculated with 9 mL of a rhizobial suspension (OD_600_ = 0.1) (3 mL per seedling) at 10 DPI. NaHS was the H_2_S donor (Zou et al. 2020). Seedlings in the H, GH, GI and GIH groups were watered with 15 mL of NaHS (100 μM) and IAA solution (100 μM) (5 mL per seedling) every 3 days until harvest, and seedlings from the other two groups were watered with double‐distilled water instead. Furthermore, 50 mL of sterile N‐free nutrient solution was added to each bag every 7 days to maintain steady humidity and ionic concentration. Seedlings from each treatment were harvested every 8 days for 10 days after inoculation, and half of the samples were dried to constant weight for dry matter determination, while the other half was immediately frozen in liquid N and stored at −80°C.

### 4.2 Shoot Dry Weight and Root Dry Weight Measurements

The shoots and roots of each of the three plants were placed separately in envelopes and then in an oven at 65°C for 48 h and weighed by an electronic analytical balance (Mettler Toledo) to obtain the dry weight (DW) of shoots and roots. This experiment was repeated with four *R. pseudoacacia* seedlings as biological replicates.

### 4.3 Chlorophyll Content, Total Nitrogen, NH_4_ ^+^‐N and NO_3_ ^−^‐N Contents Measurements

The chlorophyll contents of the leaves were identified using a chlorophyll metre (SPAD‐502 Plus, Konica Minolta). In total, 10 leaves from 10 seedlings per treatment were measured at 10:00 AM every 8 days until harvest. This experiment was repeated three times with biological replicates.

The Kjeldahl method measured the N contents with slight modifications (Corti et al. 2008). First, 0.2 g of dry sample was ground into powder and placed into a digestive tube. Then, 5 mL of concentrated H_2_SO_4_ was added, followed by shaking and mixing. Subsequently, the mixture was digested in a microwave digestion system (Labtec Line) at 365°C, and seven to eight drops of 30% H_2_O_2_ were added every 30 min. This was repeated three times until the digestion solution changed from black to transparent. Finally, the digestion solution was diluted to a constant volume with distilled water. Afterward, the determination was made using an automatic Kjeldahl apparatus (Kjeltec 8400). The N content was calculated as the DW based on the results.

For NH_4_^+^‐N determination, a series of ammonia standard solutions was used to prepare a standard curve. Dried plant material (0.05 to 0.1 g) was ground in 5 mL of 10% acetic acid, and double distilled water was added to 50 mL. A 2 mL aliquot of ammonia standard and 2 mL of supernatant were added into 0.1 mL of 1% ascorbic acid, and 3 mL of ninhydrin solution containing 50 mM ninhydrin, 10% propanol, 30% butanol and 60% ethanediol was added and mixed. The absorbance at 570 nm (A_570_) was measured and compared with the standard curve to estimate the NH_4_^+^‐N content.

For NO_3_^−^‐N determination, a series of standard KNO_3_ solutions was used to prepare a standard curve. Dried plant material (0.05 to 0.1 g) was ground in liquid N and boiled in 1 mL of double distilled water for 30 min; 0.1 mL of supernatant was added to 0.4 mL of 5% salicylic acid sulphuric acid (5 g salicylic acid dissolved in 100 mL 98% concentrated sulphuric acid). After 20 min at 25°C, 9.5 mL of 8% NaOH solution was added, followed by measuring the absorbance at 410 nm (A_410_), which was compared with the standard curve to estimate the NO_3_^−^‐N concentration.

### 4.4 Nodule Number and Nitrogenase Activity Determination

The number of nodules was recorded at the growth of 7, 10, 14, 18, 21, 26, 28, 34, 35, 42 and 50 DPI of *R. pseudoacacia* seedlings. Fifteen plants were used as a replicate, and three replicates were conducted in the experiment. Nitrogenase activity was quantified with slight modifications using the acetylene reduction method (Sloger and van Berkum 1988). Fresh *R. pseudoacacia* nodules were transferred into a 5 mL rubber‐capped airtight glass bottle filled with acetylene and air (1:100, vol/vol). The bottles were incubated at 28°C for 3 h, and ethylene content was determined using a gas chromatography system (Agilent Technologies). Standard curves generated using pure ethylene standards were employed to calibrate the gas chromatography measurements.

### 4.5 Infection Event Assay

The *M. amorphae* CCNWGS0123 strain harbouring the enhanced green fluorescence protein encoded on the pMP2444 plasmid was employed. The plasmid was incorporated into *M. amorphae* CCNWGS0123 by triparental conjugation through the assistant plasmid pRK2013 (Nishikawa et al. 2008). We defined the 2 cm segment below the root–hypocotyl junction as the infection zone, as described by Zou et al. (2019), considering that most nodules appeared in this part of the root. All the lateral roots from this segment were cut and observed, and typical infection events, including the number of root hair curling and primordium infection thread, were determined at 1 to 7 DPI using a BX53 fluorescence microscope (Olympus). Nodules were collected at 14, 21, 28 and 35 DPI, washed with sterile water, and stored in phosphate‐buffered saline (PBS). The GFP‐labelled rhizobial colonies were sectioned into 80 μm thick slices using a vibratome, and colonisation was observed under a fluorescence microscope. The above experiments were repeated four times with biological replicates. The GFP‐labelled rhizobial colonies were analysed and quantified using ImageJ software (National Institutes of Health) following a standardised workflow (Mela et al. 2022; Schindelin et al. 2012).

### 4.6 H_2_S Visualisation Detection Using Fluorescent Probe in Nodules

Freshly harvested nodules were embedded in 6% agarose, according to the methodology established by Wu et al. (2012). A vibratome (VT1200S, Leica) prepared nodule sections approximately 80 μm thick. Subsequently, they were immersed in a HEPES‐NaOH buffer (20 mM, pH 7.4) containing 5 mM of the specific H_2_S fluorescent probe, 7‐azido‐4‐methylcoumarin (AzMc, Sigma‐Aldrich) for 30 min in a dark environment, followed by three washing steps. Imaging the samples was performed using a confocal microscope (Andor Technology) with an excitation wavelength of 365 nm and an emission wavelength of 450 nm. The fluorescence intensity was analysed and quantified using ImageJ software, following a standardised workflow (Mela et al. 2022; Schindelin et al. 2012; Schroeder et al. 2021). A total of 15 nodule sections were analysed, with three sections obtained from each of the five nodules derived from different plants per treatment.

### 4.7 Light Microscopic Observation of Nodule Structure

Nodules were harvested from plants inoculated with rhizobia at 10, 18, 26 and 34 DPI. Representative nodules were chosen for the following analyses. The procedure of nodule structure observations was carried out according to the study of Chou et al. (2015). Nodules were fixed with formalin:acetic acid:alcohol stationary liquid (70% ethanol:methanol:acetic acid = 16:1:1, vol/vol/vol) for 3 days at 4°C. Fixed nodules were then dehydrated with a graded ethanol series of 50% (30 min), 80% (1 h), 95% (1 h) and 100% (1 h × 2), and every step was conducted twice at room temperature. After that, nodules were treated with 1% sarranine (vol/vol) and infiltrated with xylene:ethanol (1:1), then 100% xylene and each step was repeated twice at room temperature for 1 h, followed by infiltrating nodules with liquid filtered paraffin at 56°C for 2 days, including four paraffin changes. In the last place, nodules in solidified paraffin were cut into 8‐μm slices on a microtome. Slices containing the whole nodule structure were then deparaffinised with xylene (1 h), xylene:ethanol (50 min), a descending ethanol series (100%, 95%, 80%, 70%, 50% and 30%, 10 min each) and distilled water (5 min). After deparaffinisation, nodule slices were stained with 0.5% toluidine blue for 5 min. Finally, after treatment with distilled water, 95% ethanol and xylene, nodule structures were observed under an optical microscope (Olympus) and images were captured.

### 4.8 Electron Microscopy Observation of Nodule Micromorphology

Nodules harvested at 10, 18, 26 and 34 DPI were observed using a transmission electron microscope (FEI) to detect the micromorphological differences between G and GH groups. Sample preparation was carried out as described by Chou et al. (2015) with slight modifications. Nodules were cut into tiny slices, prefixed in 4% glutaraldehyde, washed with 0.1 M pH 6.8 phosphate buffer, and post‐fixed in 1% osmium tetroxide. After at least 3 h of fixation, nodules were dehydrated in an ascending ethanol series and embedded in LR White resin. Finally, thin sections were excised from the embedded samples using an ultramicrotome (EM TRIM2, Leica Microsystems Inc.) with a glass knife, and ultrathin sections were mounted on copper grids for transmission electron microscopy examination.

### 4.9 Total RNA Isolation, Reverse Transcription and Gene Expression Analysis

Total RNA isolation was performed using the MiniBEST Plant RNA Extraction Kit (TaKaRa) according to the manufacturer's instructions. RNA integrity was examined by 1% agarose gel electrophoresis. RNA concentration was determined using an Epoch microplate spectrophotometer (BioTek). Reverse transcription was conducted using PrimerScript RT master Mix (TaKaRa) as suggested by the manufacturer. Quantitative PCR (qPCR) was carried out with a Quantstudio 6 Flex real‐time PCR system (Thermo Fisher) and SYBR Premix Ex Taq II (TaKaRa). The qPCR programmes were described in Table S3. Primers used for expression assay were designed with Primer Premier 5.0 software (Premier Biosoft International) according to the coding sequences (CDS) of the respective genes (Wang et al. 2019). Target fragments amplified by the primers were sequenced and checked with agarose gel to ensure the accuracy of the qPCR. The sequences of the primers used in the present study are listed in Table S4. The comparative threshold cycle (*C*t) method was used to determine the relative amount of gene expression. The 18S ribosomal RNA gene and *ACTIN and GAPDH* were used as endogenous control genes to normalise the quantification of transcript abundances in *R. pseudoacacia* and *M. amorphae*, respectively. The mRNA transcriptional abundance value of genes was expressed as 2^−ΔΔCt^ (Livak and Schmittgen 2001). RT‐qPCR was conducted with three biological replicates and three technical replicates.

### 4.10 Analysis of the Transcriptome

RNA was extracted from fresh plant roots and nodules of Control, H, G and GH at 34 DPI. RNA quality was assessed using agarose gel electrophoresis, while concentration and purity were determined using Nanodrop. Additionally, RNA integrity was evaluated using Agilent 2100. Afterward, clean data sets were obtained by filtering out low‐quality reads containing N bases or adapter‐related sequences. All subsequent data analysis was performed based on these valid data sets. Furthermore, gene expression levels were analysed by calculating the read count for each sample relative to each gene and converting it into FPKM values (Li and Dewey 2011). Among RNA‐seq techniques, FPKM is currently the most widely used method for estimating gene expression levels (Trapnell et al. 2010). For samples with biological duplicates, differential gene expression between the two samples was analysed by using DESeq R package (Anders and Huber 2010; Wang et al. 2010). Genes with a *p* value < 0.05 were considered differentially expressed genes. In this study, transcriptomic analysis of *R. pseudoacacia* roots is presented in Figure 5, with the corresponding raw data provided in Table S1. Transcriptomic analysis of nodules is shown in Figure S5, and the associated raw data are provided in Table S2.

### 4.11 Alignment of GH3.1 Sequences and Construction of GH3.1 in *R. pseudoacacia* Evolutionary Tree

To investigate the evolutionary relationships of the GH3.1 protein in *R. pseudoacacia* , sequence alignment was conducted using the DNAMAN software (https://www.lynnon.com/dnaman.html), which allows for accurate alignment of amino acid sequences and facilitates the identification of conserved domains. Multiple sequences of GH3.1 from *R. pseudoacacia* were aligned with those from related species to assess sequence similarity and evolutionary conservation. The aligned sequences were further analysed to identify key functional motifs and evolutionary signatures within the GH3.1 protein family. The evolutionary tree was constructed using the MEGA 64 software, employing the neighbour‐joining (NJ) method to infer the phylogenetic relationships. Bootstrapping with 1000 replicates was applied to assess the confidence levels of the tree branches. The final phylogenetic tree was visualised and analysed to explore the divergence and evolutionary history of GH3.1 across different plant species, with a specific focus on *R. pseudoacacia* and its closely related taxa.

### 4.12 Persulfidation Assays

The coding sequence of GH3.1 in *R. pseudoacacia* was cloned into the pET15a‐His vector using the ClonExpress II One Step Cloning Kit (Vazyme). Site‐directed mutagenesis was conducted using the Mut Express II Fast Mutagenesis Kit (Vazyme). Recombinant proteins were expressed in *Escherichia coli* BL21 cells and purified with an Ni‐NTA prepacked gravity column (Sangon Biotech). The protein extracts were analysed via 10% SDS‐PAGE and then stained with Coomassie Brilliant Blue to assess purity. After purification, the proteins were digested with trypsin (Promega), and the resulting peptides were subjected to persulfidation analysis using liquid chromatography–tandem mass spectrometry (LC–MS/MS) (Fusion Lumos, Thermo Fisher) (Shen et al. 2020). Persulfidated proteins were detected using a modified tag‐switch technique (Aroca et al. 2017). Biotin‐labelled proteins were identified using an anti‐biotin antibody (Abcam), and total protein quantification was conducted using an anti‐His antibody (Abbkine).

### 4.13 Hormone Quantification by Ultra‐Performance Liquid Chromatography‐Electrospray Ionisation Tandem Mass Spectrometry (UPLC‐ESI‐MS/MS)

Fresh roots or nodules were pulverised to a fine powder in liquid nitrogen. Approximately 50 mg of this powder and a mixture of internal standards were extracted with 1 mL of ethyl acetate (Tu et al. 2024). The mixture was vortexed for 10 s and sonicated for 20 min at 4°C. Following centrifugation at 12,000 *g* for 3 min at 4°C, the supernatant was collected and evaporated to dryness using a vacuum concentrator (LABACONCO) at 30°C. The resulting dry residues were then reconstituted in 200 μL of 70% (vol/vol) methanol and filtered through a 0.22 μm PVDF syringe filter (Millipore) before UPLC analysis. Sample injection was carried out on a UPLC system connected to a triple‐quadrupole mass spectrometer (XEVO TQ‐S, Waters).

Analysis was performed on an Acquity BEH C18 column (2.1 × 100 mm, 1.7 μm, Waters) maintained at 40°C. The mobile phases used were A (5 mM ammonium acetate) and B (methanol), with a gradient elution profile: from 5% B to 20% B over 0–2.5 min, ramping to 45% B at 3 min, then to 100% B from 4 to 5 min, and returning to the starting conditions, which were maintained for an additional 3 min at a flow rate of 0.3 mL/min. All hormones were detected in the MRM scanning mode, with quantification achieved via calibration curves after matrix effect correction, comparing ion peak intensities to corresponding internal standards. The raw mass data were processed using MassLynx software (v. 4.1, Waters), and quantification was further analysed with the TargetLynx tool in MassLynx and Microsoft Excel.

### 4.14 GH3.1 Enzymatic Activity In Vitro Assay

The GH3.1 protein was expressed in *E. coli* BL21 using the pET15a‐His vector. Bacterial cultures were incubated in Luria Bertani medium at 37°C for 3 h until an OD_600_ of approximately 0.6 was achieved; then induced with 0.5 mM isopropyl‐β‐D‐thiogalactopyranoside (IPTG) at 16°C. After induction, cells were harvested via centrifugation; then resuspended in lysis buffer containing 50 mM Tris–HCl (pH 8.3), 500 mM NaCl, 20 mM imidazole and 1 mM PMSF. Protein purification was performed using the AKTA pure system (GE Healthcare) following the manufacturer's instructions. After purification, GH3.1 protein was concentrated, flash‐frozen using liquid nitrogen and stored at −80°C. The IAA‐amido synthetase assay was conducted with a reaction mixture that included 50 mM Tris–HCl (pH 8.0), 2 mM MgCl_2_, 2 mM ATP, 10 mM Asp, 0.15 mM varying concentrations of IAA (ranging from 200 to 1000 μM), and incubated at 25°C for 30 min (Ke et al. 2024). The production of IAA‐Asp/IAA‐Glu was monitored using UPLC‐MS.

### 4.15 Statistical Analysis

At least three replicates were measured for the physiological and biochemical analyses. Statistical analyses of the time‐course experiments were performed using repeated measurements of a general linear model procedure in SPSS v. 22.0 (SPSS Inc.). A one‐way analysis of variance was adopted for significant differences in the histogram, and the data were expressed as means of replicates plus their standard errors. Besides, the statistical analysis of multiple groups was tested using the Tukey test at the significance level of *p* < 0.05. Statistical significance was determined using one‐way ANOVA followed by Tukey's honest significant difference (HSD) test for pairwise comparisons. Uppercase letters indicate significant differences among stages within the same treatment, whereas lowercase letters indicate significant differences among treatments at the same stage.

## Author Contributions

Weiqin Zhang was responsible for manuscript writing and physiological and biochemical experiments; Huaping Cheng, Shiming Wen, Bingyu Suo and Xiaowu Yan were responsible for cultivation and harvest of *R. pseudoacacia* ; Wuyu Liu was responsible for vector construction; Juan Chen was responsible for manuscript revision; Juan Chen and Gehong Wei were responsible for experimental guidance, manuscript polishing and financial support.

## Conflicts of Interest

The authors declare no conflicts of interest.

## Supporting information


**Figure S1:** Fluorescence intensity analysis of GFP‐labelled *Mesorhizobium* colonisation in nodules of *Robinia pseudoacacia* . Quantification of GFP fluorescence intensity in G ( *M. amorphae* GS0123 inoculation), and GH ( *M. amorphae* GS0123 + 100 μM NaHS) groups from Figure 2c using ImageJ software. Nodule samples were collected at 14, 21, 28 and 35 days post‐inoculation (DPI). Values are means ± SE from at least three biological replicates. Bars with different letters indicate significant differences (*p* < 0.05).

**Figure S2:** Expression analysis and verification of nodulation genes in early inoculation period at different time points. Roots were collected from Control (uninoculated), H (uninoculated+100 μM NaHS), G and GH groups at 0.5, 1, 3, 5, 7 and 21 DPI. Analysed genes include: *NPL*, *ENOD40*, *SYMRK*, *NUO133*, *NUP85*, *LHK*, *Cerberus*, *CCaMK*, *CYCLOP*, *Castor* and *NIN*. The colour gradient from blue to red indicates significant differences (*p* < 0.05). Each value represents the mean ± SE (*n* = 3).

**Figure S3:** Analysis of fluorescence intensity in the G and GH roots and nodules of *Robinia pseudoacacia* . (a) Quantification of H_2_S fluorescence intensity in G and GH groups from Figure 3a using ImageJ. (b) Determination of H_2_S content by biochemical assay. Roots and Nodules were collected at 7, 14, 21, 28 and 35 DPI. Values are means ± SE from at least three biological replicates. Bars with different letters indicate significant differences (*p* < 0.05).

**Figure S4:** Quantitative analysis of nodule zonation and bacteroid abundance in nodules. Nodules were collected from G and GH groups at 10, 18, 26 and 34 DPI. (a) Quantification of zonal area based on paraffin sections shown in Figure 4a. (b) Quantification of bacteroid numbers with different sizes based on transmission electron microscopy images in Figure 4b. Uppercase letters indicate significant differences among zones within the same treatment, whereas lowercase letters denote significant differences between treatments within the same zone (*p* < 0.05).

**Figure S5:** Differential analysis of transcription levels of the *Mesorhizobium amorphae* GS0123 strain in indeterminate nodule. Nodules were collected from G and GH groups at 34 DPI. (a) Venn diagram of differentially expressed genes. (b) Enrichment pathways influenced by H_2_S. (c) Clustering heat map of genes regulated by H_2_S‐induced expression in the *M. amorphae* GS0123 strain.

**Figure S6:** qRT‐PCR verification of *Robinia pseudoacacia* roots transcriptome enrichment differential genes. Roots were collected from Control, H, G and GH groups at 0.5, 1, 3, 5, 7 and 21 DPI. (a) Heat map of enrichment for differential gene expression in metabolic pathways. (b) Heat map of enrichment for differential gene expression in biosynthesis of secondary metabolites. (c) Heat map of enrichment for differential gene expression in plant hormone signal transduction. The colour gradient from blue to red indicates significant differences (*p* < 0.05). Each value represents the mean ± SE (*n* = 3).

**Figure S7:** Analysis of fluorescence intensity in the GI and GIH roots and nodules of *Robinia pseudoacacia* . (a) Quantification of H_2_S fluorescence intensity in GI ( *Mesorhizobium amorphae* GS0123 + 100 μM IAA), and GIH ( *M. amorphae* GS0123 + 100 μM IAA + 100 μM NaHS) groups from Figure 7a using ImageJ. (b) Determination of H_2_S content by biochemical assay. Roots and Nodules were collected at 7, 14, 21, 28 and 35 DPI. Values are means ± SE from at least three biological replicates. Bars with different letters indicate significant differences (*p* < 0.05).

**Figure S8:** Expression analysis of nodulation‐related genes in nodule during nodule development and senescence. Nodules were collected from Control, H, G and GH groups at 18, 26, 34, 42 and 50 DPI. Analysed genes include: *SYMRK*, *CCaMK*, *Castor*, *CYCLOP*, *NUP85*, *NUP133*, *Cerberus*, *LHK*. Uppercase letters indicate significant differences among stages within the same treatment, whereas lowercase letters indicate significant differences among treatments at the same stage (*p* < 0.05).

**Figure S9:** Expression quantitative analysis of symbiotic nodulation related genes in *Robinia pseudoacacia* nodule. Nodules were collected from Control, H, G and GH groups at 18, 26, 34, 42 and 50 DPI. Analysed genes include: *nifH*, *nodC*, *zheng44*, *zheng45*, *zheng190*, *zheng273*. Uppercase letters indicate significant differences among stages within the same treatment, whereas lowercase letters indicate significant differences among treatments at the same stage (*p* < 0.05).

**Table S1:** Differentially expressed transcriptome genes in *Robinia pseudoacacia* roots.

**Table S2:** Differentially expressed transcriptome genes in *Robinia pseudoacacia* nodules.

**Table S3:** Procedures of dsDNA synthesis used in RT‐qPCR.

**Table S4:** Primers used in this study.

## Data Availability

The data that supports the findings of this study are available in the Supporting Information of this article.
