# Supplementary figures and images for: H 2 S‐Mediated GH3.1 Persulfidation Regulates IAA Homeostasis to Enhance Nodulation Formation and Nitrogen Fixation in Robinia pseudoacacia
Source: Mol Plant Pathol. 2025 Oct 3;26(10):e70145. doi: 10.1111/mpp.70145 (PMC12495139; doi:10.1111/mpp.70145)

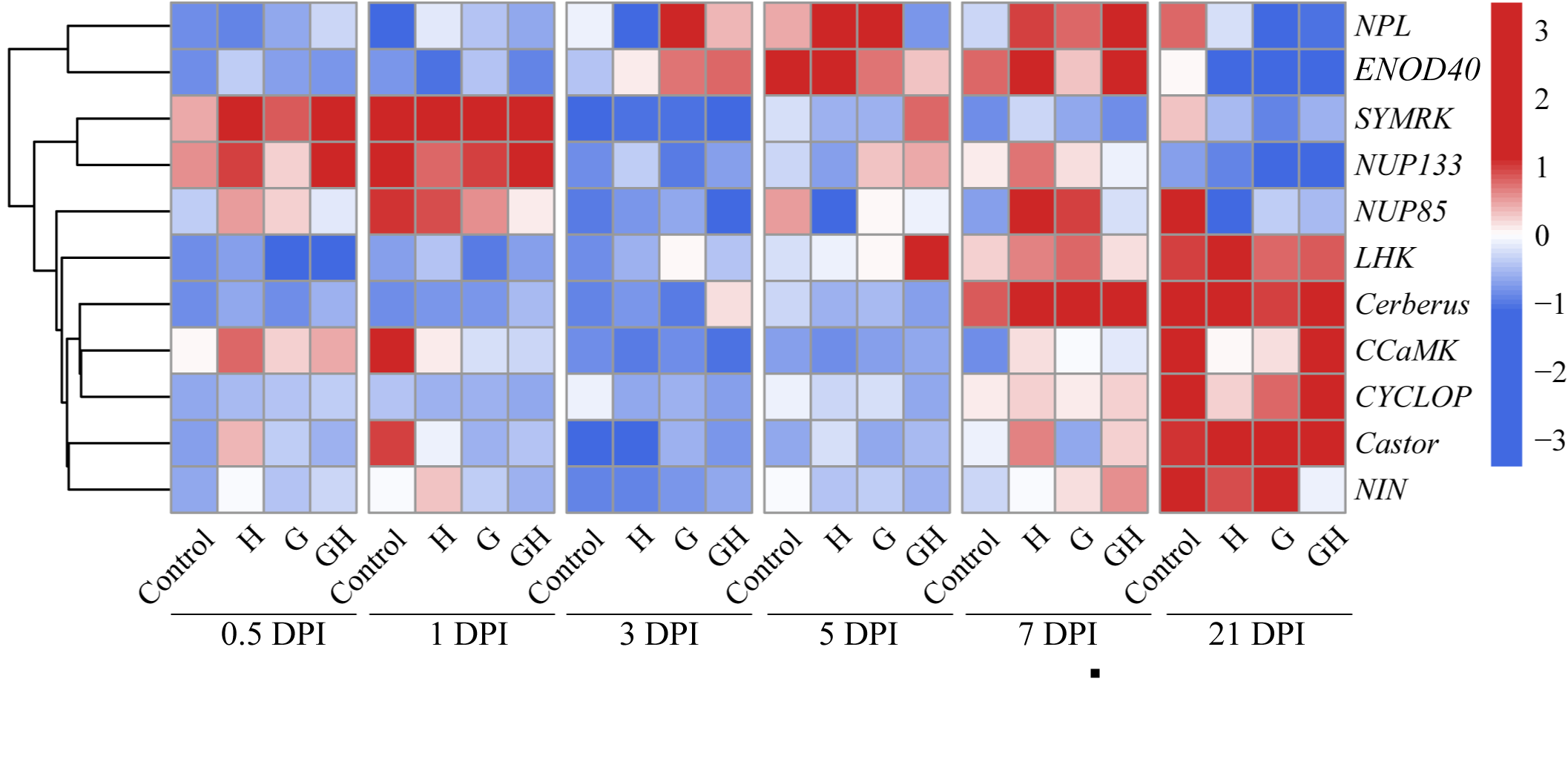

Supplement: Supplementary file 2 — Figure S2: Expression analysis and verification of nodulation genes in early inoculation period at different time points. Roots were collected from Control (uninoculated), H (uninoculated+100 μM NaHS), G and GH groups at 0.5, 1, 3, 5, 7 and 21 DPI. Analysed genes include: NPL, ENOD40, SYMRK, NUO133, NUP85, LHK, Cerberus, CCaMK, CYCLOP, Castor and NIN. The colour gradient from blue to red indicates significant differences (p < 0.05). Each value represents the mean ± SE (n = 3). [file MPP-26-e70145-s003.tif]

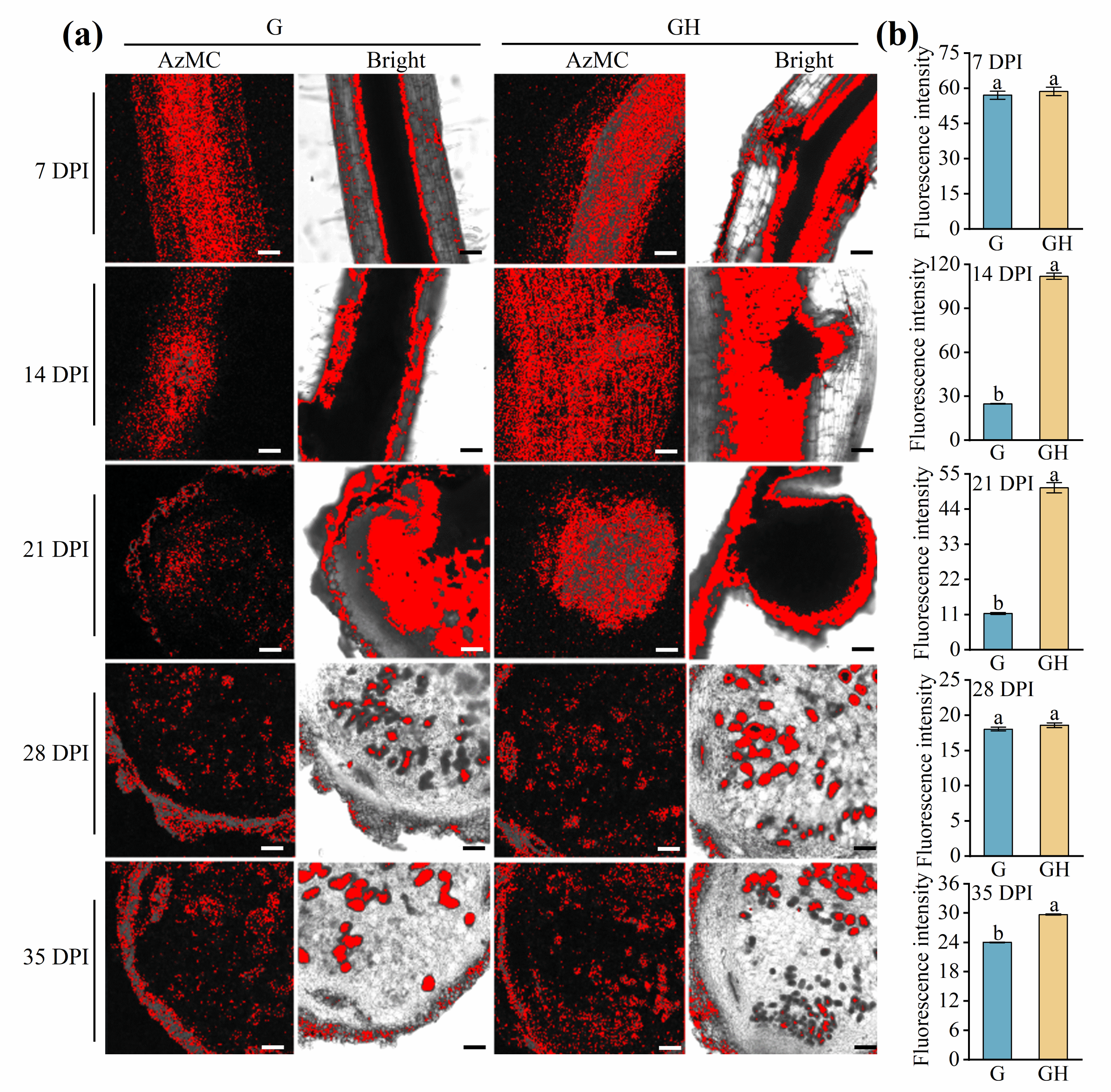

Supplement: Supplementary file 3 — Figure S3: Analysis of fluorescence intensity in the G and GH roots and nodules of Robinia pseudoacacia . (a) Quantification of H2S fluorescence intensity in G and GH groups from Figure 3a using ImageJ. (b) Determination of H2S content by biochemical assay. Roots and Nodules were collected at 7, 14, 21, 28 and 35 DPI. Values are means ± SE from at least three biological replicates. Bars with different letters indicate significant differences (p < 0.05). [file MPP-26-e70145-s013.tif]

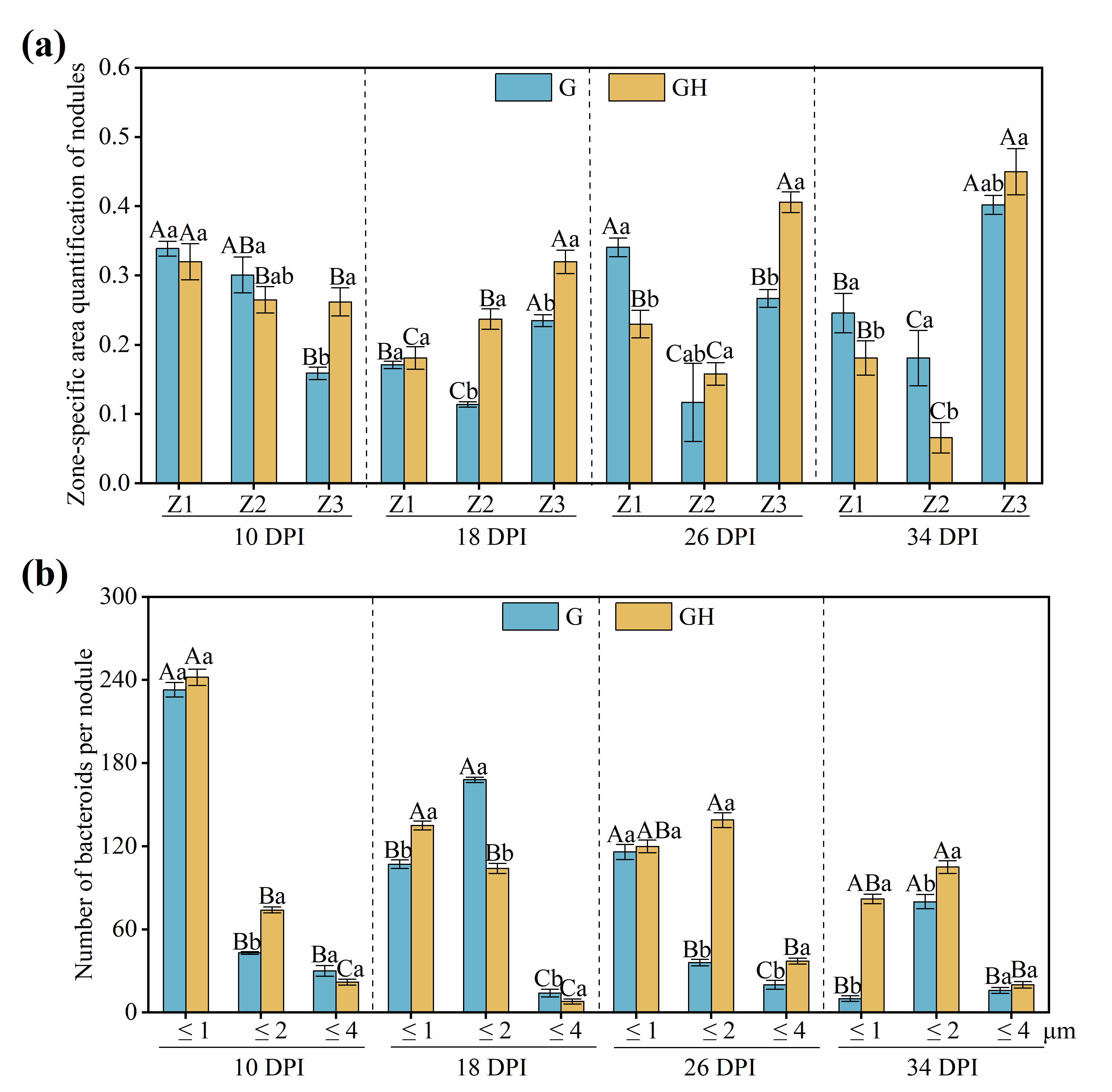

Supplement: Supplementary file 4 — Figure S4: Quantitative analysis of nodule zonation and bacteroid abundance in nodules. Nodules were collected from G and GH groups at 10, 18, 26 and 34 DPI. (a) Quantification of zonal area based on paraffin sections shown in Figure 4a. (b) Quantification of bacteroid numbers with different sizes based on transmission electron microscopy images in Figure 4b. Uppercase letters indicate significant differences among zones within the same treatment, whereas lowercase letters denote significant differences between treatments within the same zone (p < 0.05). [file MPP-26-e70145-s009.tif]

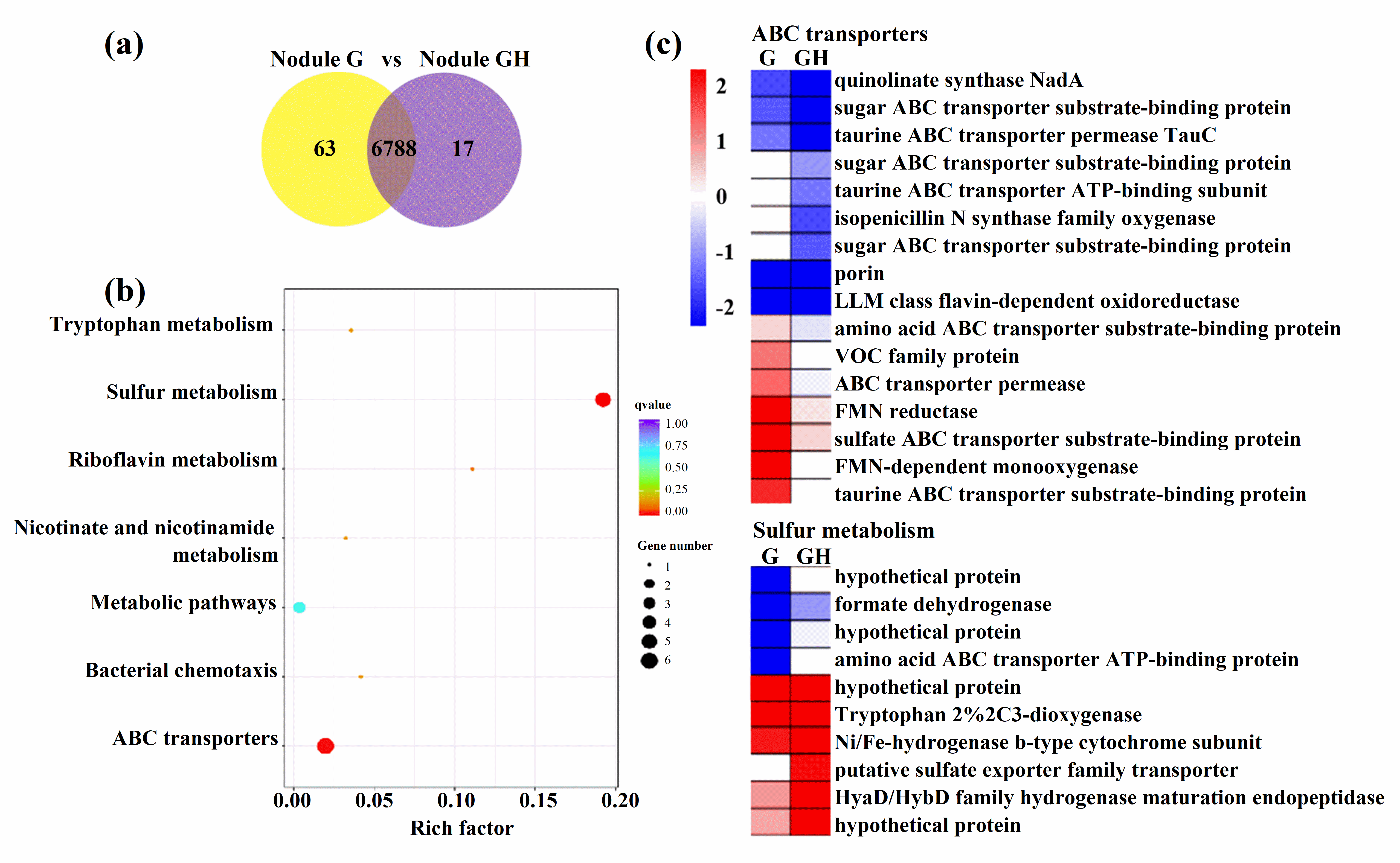

Supplement: Supplementary file 5 — Figure S5: Differential analysis of transcription levels of the Mesorhizobium amorphae GS0123 strain in indeterminate nodule. Nodules were collected from G and GH groups at 34 DPI. (a) Venn diagram of differentially expressed genes. (b) Enrichment pathways influenced by H2S. (c) Clustering heat map of genes regulated by H2S‐induced expression in the M. amorphae GS0123 strain. [file MPP-26-e70145-s005.tif]

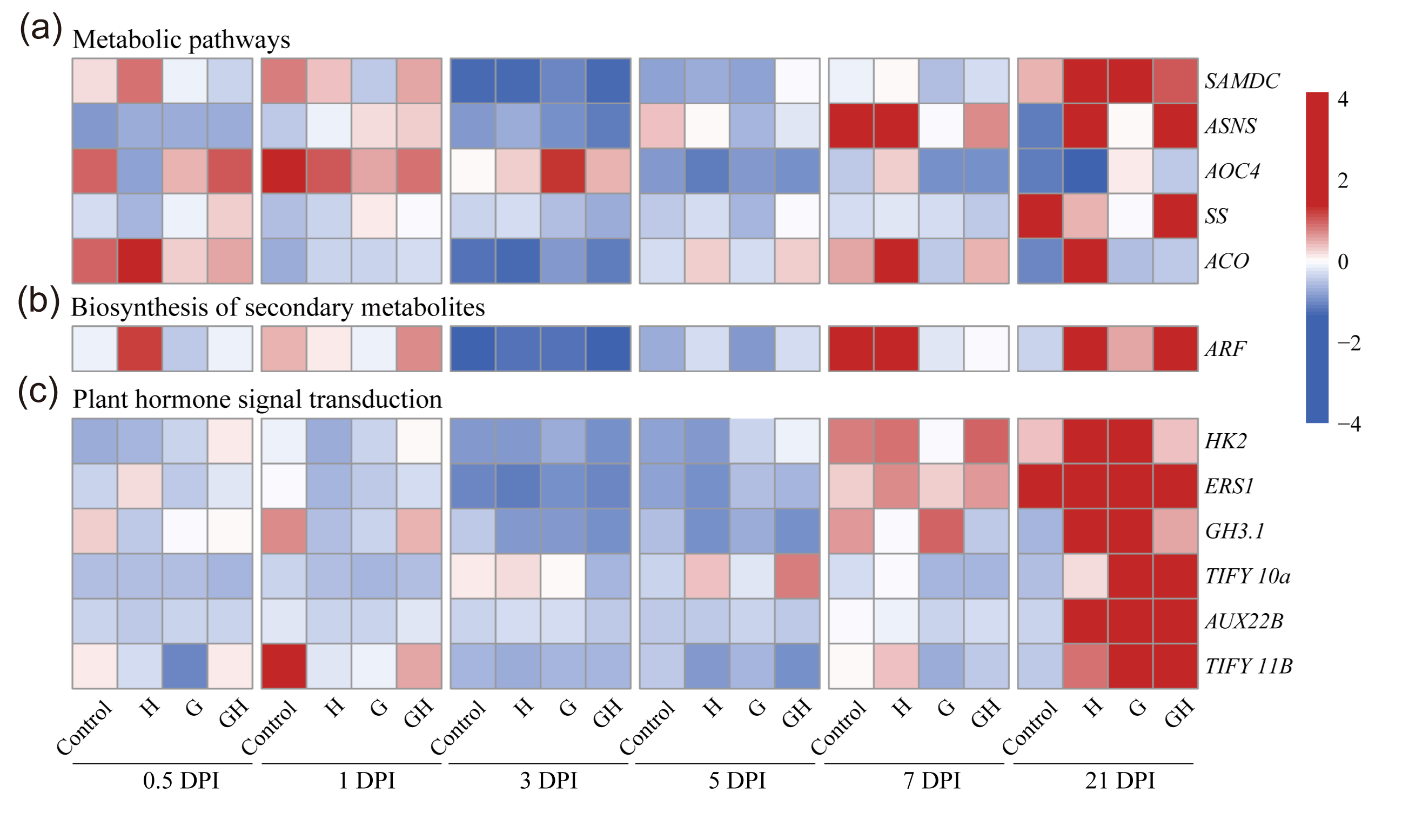

Supplement: Supplementary file 6 — Figure S6: qRT‐PCR verification of Robinia pseudoacacia roots transcriptome enrichment differential genes. Roots were collected from Control, H, G and GH groups at 0.5, 1, 3, 5, 7 and 21 DPI. (a) Heat map of enrichment for differential gene expression in metabolic pathways. (b) Heat map of enrichment for differential gene expression in biosynthesis of secondary metabolites. (c) Heat map of enrichment for differential gene expression in plant hormone signal transduction. The colour gradient from blue to red indicates significant differences (p < 0.05). Each value represents the mean ± SE (n = 3). [file MPP-26-e70145-s004.tif]

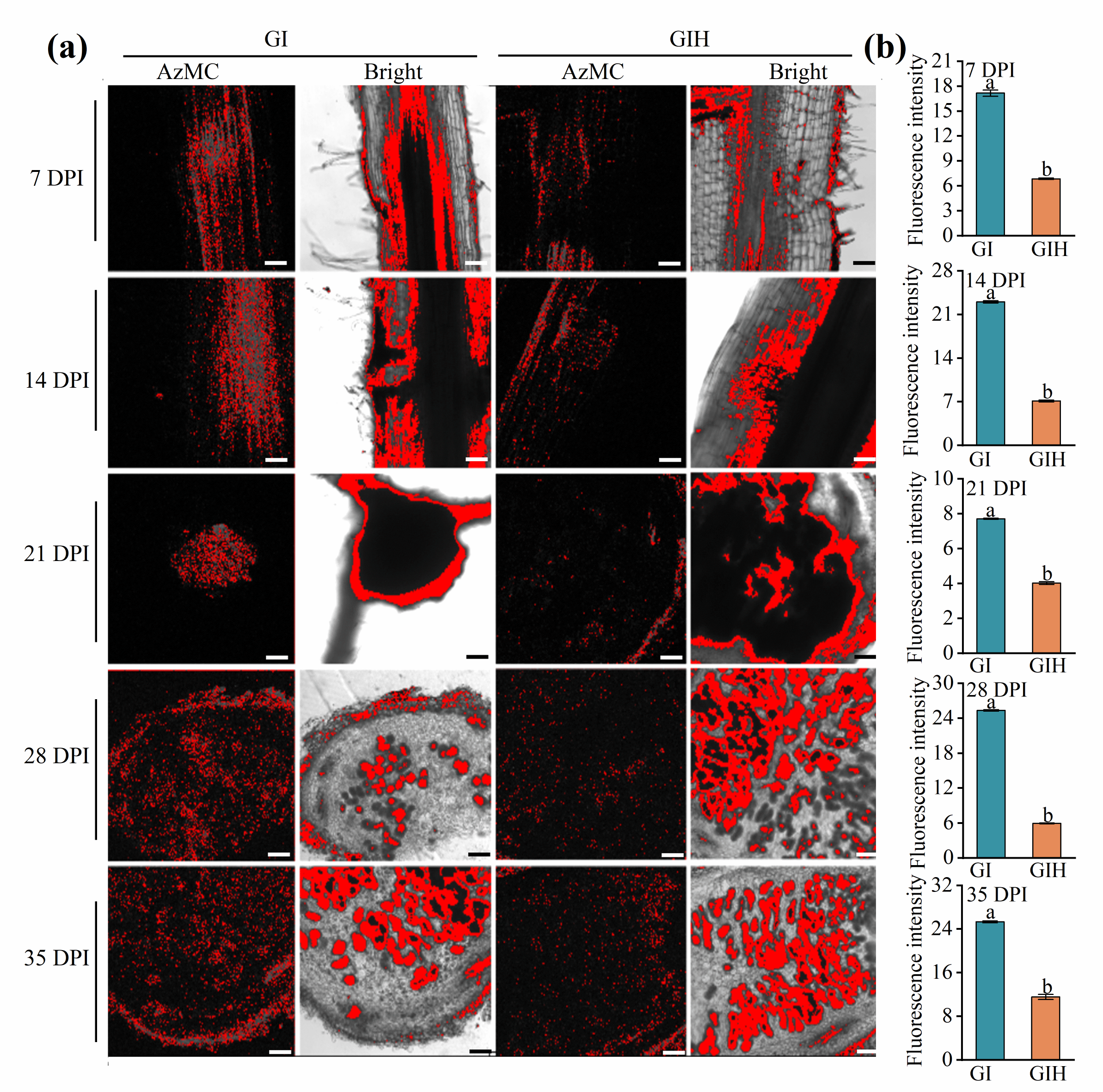

Supplement: Supplementary file 7 — Figure S7: Analysis of fluorescence intensity in the GI and GIH roots and nodules of Robinia pseudoacacia . (a) Quantification of H2S fluorescence intensity in GI ( Mesorhizobium amorphae GS0123 + 100 μM IAA), and GIH ( M. amorphae GS0123 + 100 μM IAA + 100 μM NaHS) groups from Figure 7a using ImageJ. (b) Determination of H2S content by biochemical assay. Roots and Nodules were collected at 7, 14, 21, 28 and 35 DPI. Values are means ± SE from at least three biological replicates. Bars with different letters indicate significant differences (p < 0.05). [file MPP-26-e70145-s010.tif]

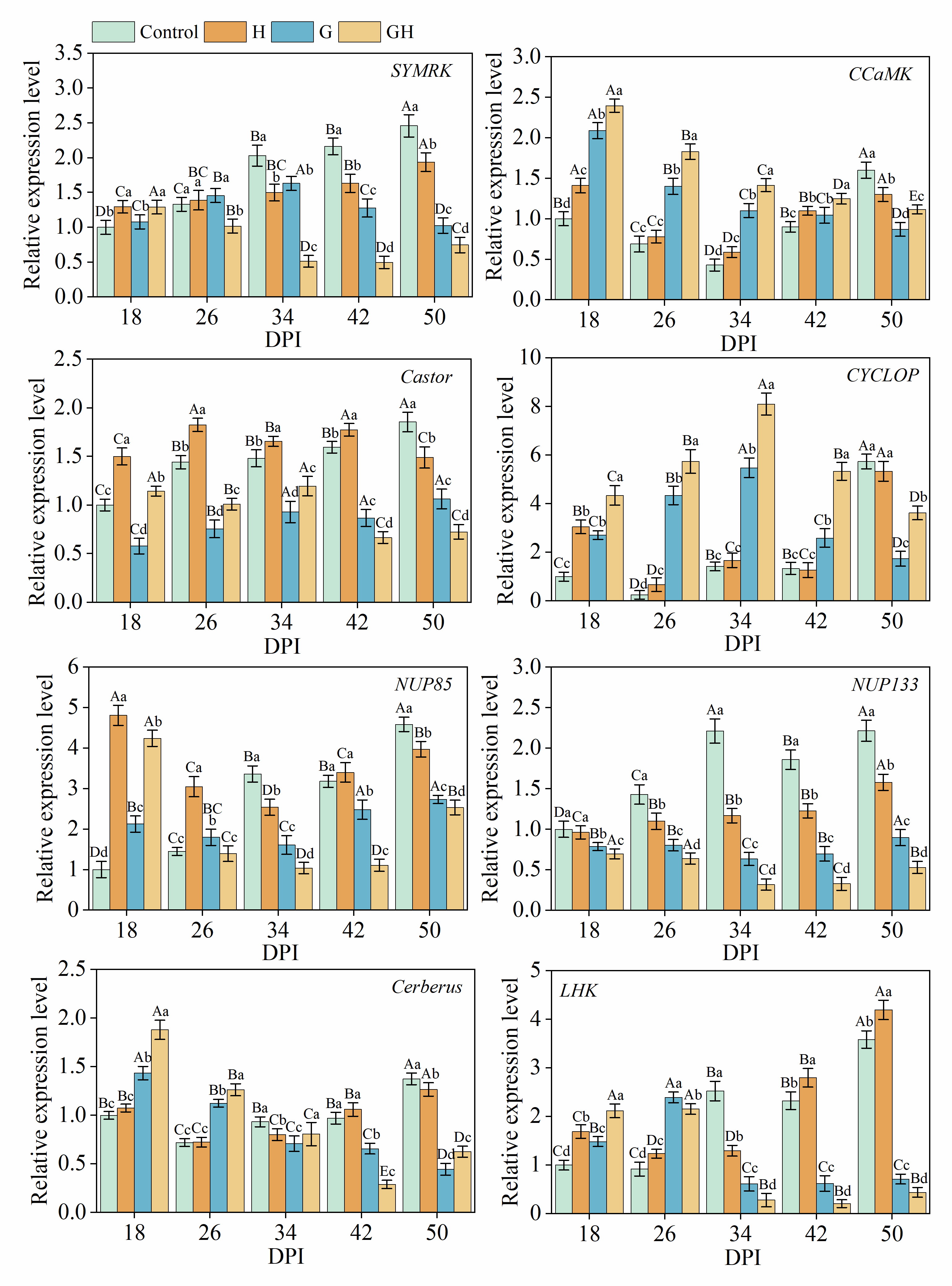

Supplement: Supplementary file 8 — Figure S8: Expression analysis of nodulation‐related genes in nodule during nodule development and senescence. Nodules were collected from Control, H, G and GH groups at 18, 26, 34, 42 and 50 DPI. Analysed genes include: SYMRK, CCaMK, Castor, CYCLOP, NUP85, NUP133, Cerberus, LHK. Uppercase letters indicate significant differences among stages within the same treatment, whereas lowercase letters indicate significant differences among treatments at the same stage (p < 0.05). [file MPP-26-e70145-s008.tif]

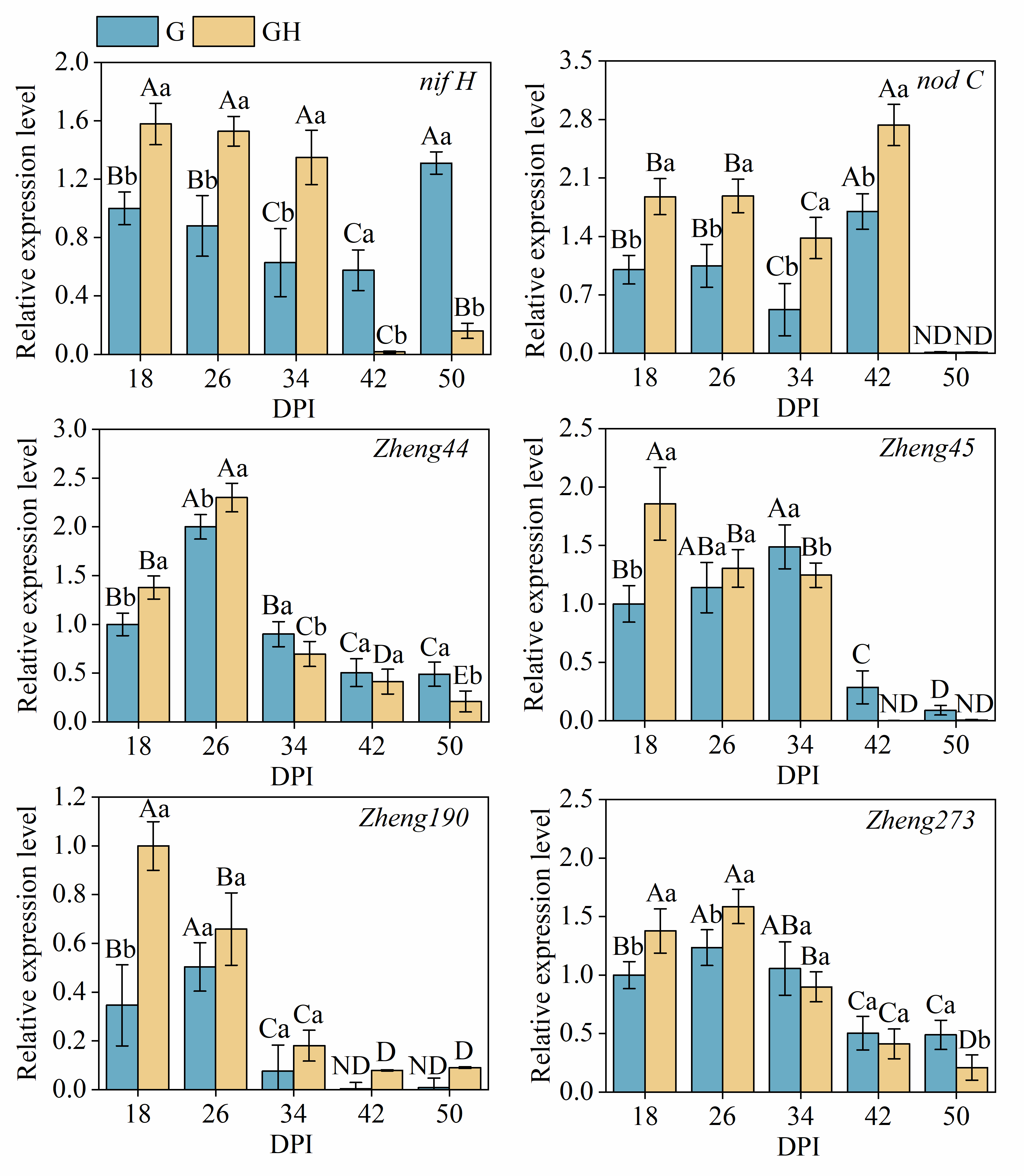

Supplement: Supplementary file 9 — Figure S9: Expression quantitative analysis of symbiotic nodulation related genes in Robinia pseudoacacia nodule. Nodules were collected from Control, H, G and GH groups at 18, 26, 34, 42 and 50 DPI. Analysed genes include: nifH, nodC, zheng44, zheng45, zheng190, zheng273. Uppercase letters indicate significant differences among stages within the same treatment, whereas lowercase letters indicate significant differences among treatments at the same stage (p < 0.05). [file MPP-26-e70145-s012.tif]
